# Ubc9‐mediated SUMOylation of Ninj1 alleviates inflammatory responses in hepatic ischaemia/reperfusion injury

**DOI:** 10.1002/ctm2.70677

**Published:** 2026-05-10

**Authors:** Kang Huang, Li Xu, Shufang Na, Yan Xu, Qiaoyun Liu, Shaojun Ye, Cong‐Yi Wang, Wei Zhou, Qifa Ye

**Affiliations:** ^1^ Zhongnan Hospital of Wuhan University Institute of Hepatobiliary Diseases of Wuhan University Transplant Center of Wuhan University National Quality Control Center for Donated Organ Procurement, Hubei Key Laboratory of Medical Technology on Transplantation, Hubei Provincial Clinical Research Center for Natural Polymer Biological Liver, Hubei Hepatobiliary Diseases Society Wuhan China; ^2^ Diabetes Research Center Qatar Biomedical Research Institute Hamad Bin Khalifa University Doha Qatar

**Keywords:** hepatic ischaemia/reperfusion, Ninj1, SUMOylation, Ubc9

## Abstract

**Background:**

Hepatic ischaemia/reperfusion (I/R) injury poses a common clinical dilemma encountered during liver transplantation (LT), characterised by substantial cellular death and inflammation reactions. Ubc9, the sole E2 conjugating enzyme of SUMOylation, has long been recognised to regulate diverse biological and pathological processes. However, its impact on I/R‐induced liver damage is yet to be elucidated.

**Methods:**

The expression levels of UBC9 in patients undergoing LT were analysed. Hepatocyte‐specific *Ubc9*‐deficient or transgenic mice were utilised in an in vivo model of hepatic I/R, alongside in vitro experiments that employed hypoxia/reoxygenation stimulation. The investigation focused on Ubc9's role in liver damage due to I/R and the underlying mechanisms through a range of phenotypic analyses and biological techniques.

**Results:**

Herein, we found that hepatic tissues from patients with LT are featured by a significant downregulation of UBC9 expression. Studies in 68 donor hepatic biopsies further demonstrated a negative correlation between UBC9 expression and liver injury in patients with LT. Similarly, murine liver I/R was coupled with an obvious decrease in Ubc9 expression. Hepatocyte deficient in *Ubc9* exacerbated liver injury in liver I/R, while *Ubc9*‐overexpression showed the opposite phenotype. Mechanistically, Ubc9‐mediated SUMOylation of Ninj1 at lysine K103 inhibited its membrane localisation and damage‐associated molecular patterns (DAMPs) release in hepatocytes, subsequently inhibited nuclear factor‐kappa B (NF‐κB) signalling in macrophages and curtailing inflammatory cytokines production.

**Conclusions:**

These findings further suggest that Ubc9‐mediated SUMOylation of Ninj1 at lysine K103 may represent a potential therapeutic strategy for safeguarding the liver against I/R injury in clinical settings.

**Key points:**

- Ubc9 expression is downregulated in hepatocytes during hepatic ischaemia/reperfusion (I/R) injury.
- Higher UBC9 expression is associated with improved post‐liver transplantation (LT) liver function.
- Ubc9 ameliorates liver damage and inflammation responses in hepatic I/R injury.
- Ubc9‐mediated Ninj1 SUMOylation at K103 is essential for regulating the subcellular distribution of Ninj1.
- Ubc9 inhibits the release of hepatocyte‐derived damage‐associated molecular patterns (DAMPs) in a Ninj1 K103 SUMOylation‐dependent manner.

## Graphical abstract

1. Ubc9 expression is downregulated in hepatocytes during hepatic I/R injury.
2. Higher UBC9 expression is associated with improved post‐LT liver function.
3. Ubc9 ameliorates liver damage and inflammation responses in hepatic I/R injury.
4. Ubc9‐mediated Ninj1 SUMOylation at K103 is essential for regulating the subcellular distribution of Ninj1.
5. Ubc9 inhibits the release of hepatocyte‐derived DAMPs in a Ninj1 K103 SUMOylation‐dependent manner.

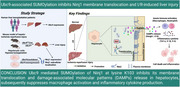

## 1 INTRODUCTION

Hepatic ischaemia/reperfusion (I/R) injury constitutes a significant pathological mechanism and is a key element in the early dysfunction or rejection of allografts, which is linked to poor prognosis following liver transplantation (LT).^1^ Despite the employment of many strategies against hepatic I/R injury, such as antioxidants or anti‐inflammatory drugs, and ischaemic preconditioning, the outcome failed to reach the expected satisfaction.^2^, ^3^, ^4^ Therefore, exploration of more efficient and safe treatment is crucial to ensure successful LT in clinical settings.

Hepatic I/R injury represents a complex and evolving phenomenon that involves various cells and diverse processes. It is marked by the progressive injuries to hepatocytes, apoptosis or necrosis, and acute inflammatory responses.^5^ During the ischaemic phase, a lack of oxygen and glucose, depletion of ATP, and disturbances in cellular metabolism can cause liver damage either directly or indirectly. During the reperfusion phase, the reestablishment of blood flow triggers the generation of reactive oxygen species (ROS), leading to further liver injury.^1^, ^6^ These damaged hepatocytes release various damage‐associated molecular patterns (DAMPs), which then stimulates inflammatory responses, including the infiltration of macrophages and neutrophils and an overproduction of inflammatory cytokines, thereby exacerbating liver injury.^1^, ^7^, ^8^, ^9^

SUMOylation refers to a modification of proteins where a small ubiquitin‐like modifier (SUMO) is covalently bonded to substrates, which in turn affects protein stability, localisation within cells and interactions between proteins.^10^, ^11^ The process of SUMOylation involves a variety of signalling molecules, including transcription factors/co‐regulators, histone‐modifying factors and metabolic enzymes.^12^, ^13^ This modification is a complex process at the participation of multiple enzymes.^14^ As a only E2 enzyme of SUMOylation, Ubc9 is crucial for proper embryonic development, and is dysregulated in numerous diseases such as cancer and ischaemia.^15^, ^16^, ^17^ However, whether and how Ubc9 regulates liver injury induced by I/R remains unclear.

Ninj1 is a membrane protein and is widely expressed in various cells.^18^ Ninj1 has two extracellular regions at the N‐ and C‐terminus, two transmembrane segments and a cytoplasmic domain.^19^ An adhesion motif located within the N‐terminal extracellular domain (26–37 amino acids) has been identified, which facilitates cell‐to‐cell adhesion.^20^ The liver expresses Ninj1 in hepatocytes and liver sinusoidal endothelial cells.^21^ Furthermore, a previous study suggested that Ninj1 promoted neutrophils infiltration and exacerbated I/R‐induced liver injury.^22^ Nevertheless, the exact mechanism through which Ninj1 mediates hepatic injury induced by I/R is still not entirely clarified.

In this study, we examined the function of Ubc9 during liver I/R injury. By utilising hepatocyte‐specific *Ubc9*‐deficient or transgenic mice, we revealed that Ubc9 ameliorated I/R‐induced hepatic inflammatory responses. Mechanistically, loss of *Ubc9* promoted damaged hepatocytes release DAMPs to activate macrophages and induce inflammatory responses. Moreover, Ubc9‐mediated Ninj1 SUMOylation to inhibit its membrane translocation, thus reducing DAMPs release following I/R challenge. Collectively, our findings indicated that Ubc9‐mediated Ninj1 SUMOylation played a crucial protective role in hepatic injury induced by I/R, highlighting its potential as a target for therapeutic strategies aimed at addressing hepatic I/R injury in clinical settings.

## 2 MATERIALS AND METHODS

### 2.1 Clinical data and samples

Pre‐LT and post‐LT hepatic samples were gathered at Zhongnan Hospital of Wuhan University. Pre‐LT hepatic samples were collected during organ procurement, and post‐LT samples were collected 2–3 h after the portal reperfusion (before the closure of the abdomen). The duration of ischaemia was determined as the interval between the donor liver's perfusion with University of Wisconsin solution and its extraction from cold storage. The clinical data of donors and recipients were collected.

### 2.2 Animals

Male C57BL/6J wild‐type (WT) mice, aged 6–8 weeks, were sourced from Wanqian Jiaxing Biotechnology Co. Ltd. The generation of hepatocyte‐specific *Ubc9* knockout (KO) (*Ubc9^HKO^*) and hepatocyte‐specific *Ubc9* transgenic (*Ubc9^Tg^*) mice was conducted as previously reported.^23^ *Ubc9^HKO^* mice were obtained by crossing *Ubc9^fl/fl^* mice with *Alb‐Cre* mice. *Ubc9^Tg^* mice were obtained by crossing *Ubc9* transgenic mice with *Alb‐Cre* mice. Genotyping based on polymerase chain reaction (PCR) methods was carried out with the primer pairs and detailed in Table S1.

### 2.3 Hepatic I/R injury model

A model of partial warm ischaemia (70% liver) was created as detailed in previous studies.^6^ After the indicated time of ischaemia, the clamp was released to allow reperfusion. Sham‐operated mice that underwent surgery experienced the same protocol but without vascular blockage. Anti‐Hmgb1 neutralising (Arigo Biolaboratories, SQab20175) antibody, at a dosage of 100 µg dissolved in 100 µL sterile phosphate‐buffered saline (PBS), was administered intravenously through the tail vein 1 h before hepatic I/R. Adeno‐associated virus (AAV‐Con and AAV‐*Ninj1* K103R) was purchased by MiaoLingBio. *Ubc9^Tg^* mice were injected via tail (6 × 10^11^ pfu in 150 µL per mouse) 2 weeks before hepatic I/R. Mice were sacrificed, and serum and tissues were promptly gathered for further analyses.

### 2.4 Cell culture

HEK293T cells (catalogue STCC10305) and AML12 cells (catalogue STCC20037) were acquired from Servicebio. HEK293T cells were cultured in medium (Servicebio, G4511) enriched with 10% foetal bovine serum (FBS) (Yeasen, 40130ES76) at 37°C in 5% CO_2_. AML12 cells were cultured in Dulbecco's modified Eagle medium (DMEM) supplemented with 10% FBS, 1% penicillin‒streptomycin, 1% ITS Media Supplement (Beyotime, C0341) and 40 ng/mL dexamethasone (OriLeaf, R22035) at 37°C in 5% CO_2_. AML12 *Ninj1* KO cells were developed utilising the CRISPR/cas9 genome editing technique (Genomeditech [Shanghai] Co., Ltd.). The plasmids (*Ninj*1 WT and *Ninj*1 K103R) were constructed by MiaoLingBio. AML12 *Ninj*1 KO cells were reconstituted with WT mouse N*inj*1 (*Ninj*1 WT) or mutant mouse *Ninj*1 (N*inj*1 K103R) cloned into pLV3‐CMV‐*Gfp*‐puro vector (MiaoLingBio), respectively. The stable transduced cells were established through 1 month of puromycin (Servicebio, G4017) selection.

### 2.5 Cell transfection

For siRNA‐mediated gene knockdown, AML12 cells were transfected with an *Ubc9* siRNA (si‐*Ubc9*) or its scramble control (si‐NC) for 48 h. siRNAs were obtained from Tsingke Biotech. For plasmid‐mediated gene overexpression, *Ubc9* plasmid, *Flag*‐tagged *Ubc9* plasmid, *Sumo1* plasmid, *Gfp*‐tagged *Sumo1* plasmid, *HA*‐tagged *Ninj1* plasmid, *HA*‐tagged *Ninj1* K103R plasmid, *HA*‐tagged *Ninj1* K114R plasmid and *HA*‐tagged *Ninj1* K103R/K114R plasmid were obtained from MiaoLingBio. Plasmid transfections were performed using the Lipo2000 reagent (Invitrogen, 11668‐019) according to the manufacture's protocol. The siRNA sequences are provided in Table S2.

### 2.6 Induction of bone marrow‐derived macrophages

Bone marrow‐derived macrophages (BMDMs) were prepared according to previously established protocols.^24^ Bone marrow cells were harvested from the femurs of WT mice (6–8 weeks old). Initially, bones underwent a wash with PBS (Servicebio, G4202) under sterile conditions. Following this, the marrow was flushed out by cutting the ends the bones and centrifuging them into PBS. Cells were plated in DMEM with 30 ng/mL mouse Macrophage Colony Stimulating Factor (M‐CSF) recombinant protein (Peprotech Inc., 315‐02‐2UG). After a 6‐day cultivation period, BMDMs were detached from the culture dishes, resuspended in fresh DMEM (Servicebio, G4511) with 30 ng/mL mouse M‐CSF recombinant protein and plated for further experiments.

### 2.7 Hypoxia/reoxygenation model

Hypoxia/reoxygenation (H/R) was conducted as detailed in previous studies.^6^ In brief, AML12 cells were maintained in DMEM medium supplemented with 10% FBS, 1% penicillin‒streptomycin, 1% ITS Media Supplement and 40 ng/mL dexamethasone at 37°C in 5% CO_2_. The medium was then replaced with DMEM devoid of serum and glucose (Servicebio, G4583), and cells were subjected to hypoxia environment (1% O_2_, 5% CO_2_ and 94% N_2_). After hypoxia challenge, the medium was again changed to DMEM enriched with 10% FBS, and then cells were returned to normoxia environment for the indicated time.

### 2.8 Isolation of primary hepatocytes and non‐parenchymal cells

Primary hepatocytes and non‐parenchymal cells (NPCs) were extracted from livers following methods outlined in earlier research.^25^ The cell suspensions were centrifuged for 2 min at 50×*g* to collect hepatocytes in the pellet. The NPCs were collected and washed using PBS buffer.

### 2.9 Measurements of liver injury

The concentrations of alanine aminotransferase (ALT) and aspartate aminotransferase (AST) in serum were evaluated utilising the ADVIA 2400 Chemistry System following the manufacture's guidelines. Hepatic injury was histologically scored according to the Suzuki's criteria.^26^

### 2.10 Migration assays

BMDM migration was conducted following previously established protocols.^8^ The supernatants from H/R‐insulted hepatocytes were harvested as conditioned medium (CM). In FBS‐free medium, BMDMs (1 × 10^4^ in 400 µL) were placed in the upper chambers of transwell systems equipped with 8‐µm pore‐size polycarbonate filters (Corning, costar3422), while 600 µL of the indicated CM was introduced into the lower chambers. After 24 h of incubation, the migrated BMDMs were counted through a digital microscope system (Leica).

### 2.11 RT‐qPCR analysis

The RT‐qPCR procedure was conducted as described earlier, utilising the ABI Prism 7500 Sequence Detection System.^27^ The relative expression levels of the target genes were determined by employing the 2^−ΔΔCt^ approach. Normalisation was carried out using the *β‐actin* gene. All primers are listed in Table S3.

### 2.12 Western blot analysis

Western blot was performed following established techniques.^27^ Proteins were isolated from tissues and cell samples, with concentrations assessed via a BCA Protein Assay Kit (Beyotime, P0012). Each sample (30 µg) was loaded onto the SDS‐PAGE gel (Epizyme, PG210), electrophoresed, blotted and incubated with indicated primary antibodies along with the corresponding secondary Horseradish Peroxidase (HRP) model for end‐stage liver disease‐conjugated antibodies. Primary antibodies were employed as follows: Ubc9 (ZENBIO, R27399), Sae1 (Abmart, TB4356), Senp1 (Abmart, TA0275S), Pias1 (Abmart, T58350S), β‐actin (Abclonal, AC026), β‐tubulin (ZENBIO, R380628), Na^+^/K^+^ATPase (Abclonal, A11683), HA (Affinity, T0050), Sumo1 (abcam, ab32058), Sumo2/3 (CST, 4971S), Bax (Abmart, T40051), Bcl2 (Abmart, T40056), Cleaved‐Caspase 3 (Abmart, TA7022), Nlrp3 (Abclonal, A24294), Cleaved‐Caspase 1 (Abclonal, A23429), Cleaved‐Caspase 8 (Abclonal, A0215), Ripk3 (CST, 95702T), p‐Ripk3 (CST, 93654T), Mlkl (CST, 37705T), p‐Mlkl (CST, 37333T), P65 (Proteintech, 10745‐1‐AP), p‐P65 (Proteintech, 82335‐1‐RR), IκBɑ (CST, 4812T), IKKβ (CST, 8943T), p‐IKKβ (CST, 2078T) and Ninj1 (HUABIO, ER61906). β‐Actin, β‐tubulin and Na^+^/K^+^ATPase were used for normalisation. Goat anti‐rabbit (Boster, BA1054) or goat anti‐mouse antibody (Boster, BA1038) was used as the secondary antibody. The intensity of each band was quantified through the use of ImageJ software.

### 2.13 Statistical analysis

All the data are represented as mean ± SEM. The data were compared using Student's *t* test, one‐way analysis of variance (ANOVA) followed with Bonferroni's multiple comparisons test when applicable. Statistical analyses for correlation were performed using Spearman's correlation. In all cases, *p <* .05 was considered as statistical significance. All statistical tests were performed by the GraphPad Prism version 10.1.2 software (GraphPad Software).

## 3 RESULTS

### 3.1 Ubc9 is closely associated with hepatic I/R injury

**FIGURE 1:**
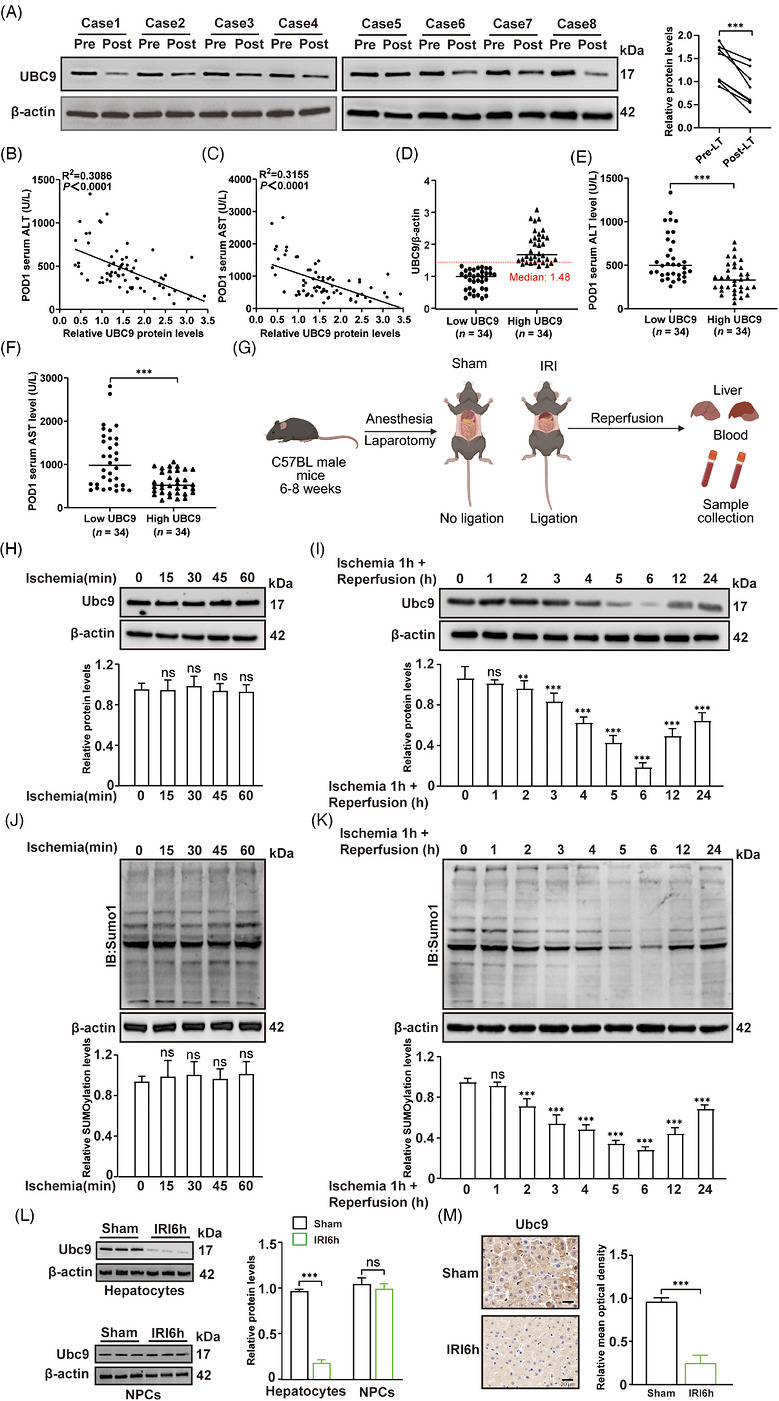
Hepatic Ubc9 expression is decreased after hepatic ischaemia/reperfusion (I/R) injury. (A) Western blot analysis of UBC9 protein expression in human liver pre‐transplant and post‐transplant tissues (*n* = 8). (B and C) The correlation analysis of pre‐transplant UBC9 protein level in human liver tissues and recipients’ serum alanine aminotransferase (ALT) (B) and aspartate aminotransferase (AST) (C) levels on the first day after liver transplantation (*n*  =  68). (D) Pre‐transplant liver samples were divided into low (*n* = 34) and high (*n* = 34) UBC9 expression groups based on the relative UBC9/β‐actin levels (cutoff = 1.48, median). (E and F) Serum ALT (E) and AST (F) in both low UBC9 (*n* = 34) and high UBC9 (*n* = 34) groups on the first day after liver transplantation (*n*  =  68). (G) Schematic of murine hepatic I/R injury experiment. (H) Ubc9 protein levels in livers from mice subjected to sham treatment or ischaemia for the indicated times (*n* = 5). (I) Ubc9 protein levels in livers from mice subjected to ischaemia for 1 h followed by reperfusion for the indicated times (*n* = 5). (J) Sumo1‐conjuncted substrates levels in livers from mice subjected to sham treatment or ischaemia for the indicated times (*n* = 5). (K) Sumo1‐conjuncted substrates levels in livers from mice subjected to ischaemia for 1 h followed by reperfusion for the indicated times (*n* = 5). (L) Ubc9 protein levels in hepatocytes and non‐parenchymal cells (NPCs) from sham and hepatic I/R (*n* = 5). (M) Immunohistochemical (IHC) analysis of the expression of Ubc9 from sham and hepatic I/R (*n* = 5). Scale bar = 20 µm. IRI6h, ischaemia for 1 h followed by reperfusion for 6 h. All the data are presented as the mean ± SEM. Paired Student's *t*‐test was used in (A). Spearman's correlation analysis was used in (B) and (C). Unpaired Student's *t*‐test was used in (E), (F) and (H–M). ^**^ *p *< .01; ^***^ *p *< .001; ns, not significant.

To explore the relationship between Ubc9 and hepatic I/R injury, we initially evaluated UBC9 expression and its association with liver injury in a cohort of human LT patients (*n* = 68). Overall, UBC9 expression levels were lower in post‐LT liver samples than that in pre‐LT liver samples (Figure 1A). Pre‐LT UBC9 expression was negative correlation with serum liver enzymes (ALT and AST) levels on postoperative day 1 (POD1) (Figure 1B, C). In addition, we classified patients into low‐ and high‐UBC9 groups utilising the median UBC9/β‐actin ratio based on pre‐LT UBC9 expression (Figure 1D). Consistently, patients in the high‐UBC9 group displayed notably decreased serum liver enzymes levels on POD1 when compared to the low‐UBC9 group (Figure 1E, F). No relationship was found between UBC9 and donor characteristics, including gender, age, pre‐procurement ALT, pre‐procurement AST, body mass index (BMI) and donation types (Table S4). Similarly, no correlation was found between UBC9 and recipient/surgical factors such as age, gender, BMI, disease aetiology, ABO compatibility, model for end‐stage liver disease (MELD) 2',7'‐Dichlorodihydrofluorescein Diacetate score, pre‐LT ALT, pre‐LT AST, cold ischaemia time (CIT), warm ischaemia time (WIT), anhepatic phase time, transplant operation time and intraoperative blood loss (Table S5). To precisely elucidate the dynamic changes of Ubc9 during hepatic I/R, we established a mouse model of hepatic I/R injury (Figure 1G). Quantitative polymerase chain reaction (Q‐PCR) results revealed no significant change in *Ubc9* mRNA levels during hepatic ischaemia and reperfusion (Figure S1A, B). And we observed no obvious changes in Ubc9 protein levels during hepatic ischaemia (Figure 1H). However, Ubc9 protein levels progressively decreased, followed by a subsequent rebound during reperfusion (Figure 1I). Ubc9 protein levels were the lowest after 1 h of ischaemia, with subsequent reperfusion lasting for 6 h (Figure 1I). Consistently, the levels of Sumo1‐conjuncted substrates and Sumo2/3‐conjuncted substrates during hepatic I/R remained similar changes (Figures 1J, K and S1C, D). While other related proteins such as Sae1, Senp1 and Pias1 remained stable throughout the study (Figure S2A).

The time point (reperfusion for 6 h) is particularly critical as it represents a peak period for acute hepatocyte injury, which is the primary focus of our investigation.^6^ Compared to the sham‐operated control, liver I/R group showed serious injury, as demonstrated by massive necrosis and significantly elevated liver enzymes (Figure S2B, C). To further clarify the cell‐type‐specific alterations of Ubc9 expression, we obtained hepatocytes and NPCs from both sham mice and hepatic I/R mice to assess Ubc9 expression. Ubc9 was dramatically decreased in hepatocytes during hepatic I/R, but its expression was obviously unchanged in NPCs during hepatic I/R (Figure 1L). These observations prompted us to focus on hepatocytes in order to investigate the role of Ubc9 during hepatic I/R. An in vitro H/R model was next employed to further validate the above results. RT‐qPCR results revealed that *Ubc9* mRNA expression was not changed significantly (Figure S2D). Ubc9, Sumo1‐conjuncted substrates and Sumo2/3‐conjuncted substrates results were similar to the results of in vivo experiments (Figure S2E–G). Immunohistochemical (IHC) staining also showed that Ubc9 was significantly decreased during hepatic I/R challenge (Figure 1M). Collectively, these data support a significant role for Ubc9 in I/R‐induced hepatic injury.

### 3.2 Ubc9 protects mice against I/R‐induced hepatic injury

**FIGURE 2:**
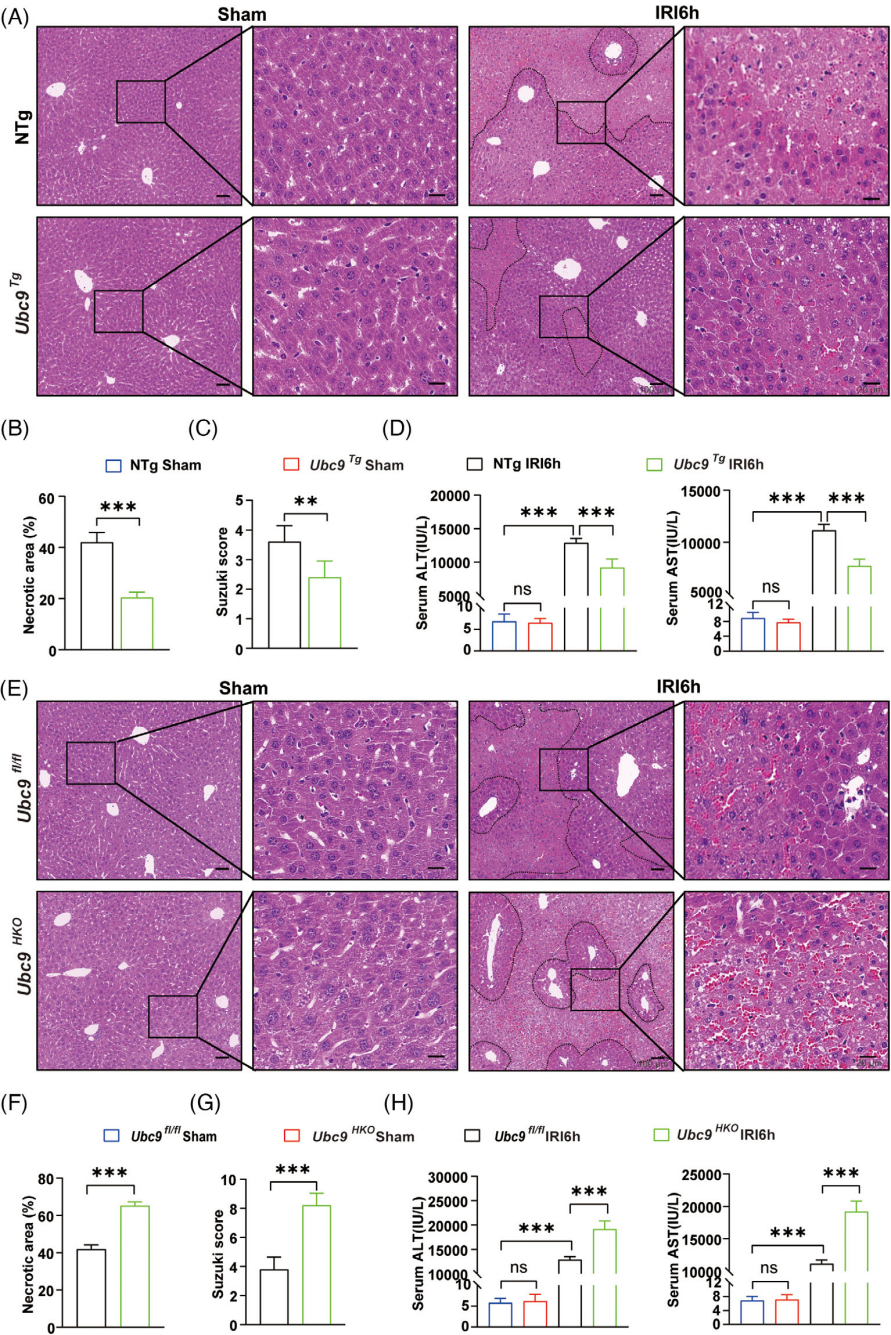
Ubc9 protects against hepatic ischaemia/reperfusion (I/R) injury. (A) Representative haematoxylin and eosin (H&E) staining images in liver tissues from NTg and *Ubc9^Tg^* mice subjected to sham or hepatic I/R (*n* = 8). Scale bar = 20 or 100 µm. (B) Quantification of necrotic areas in livers from NTg and *Ubc9^Tg^* mice subjected to sham or hepatic I/R (*n* = 8). (C) The Suzuki scores of liver sections in NTg and *Ubc9^Tg^* mice subjected to sham or hepatic I/R (*n* = 8). (D) Serum alanine aminotransferase (ALT)/ aspartate aminotransferase (AST) levels in NTg and *Ubc9^Tg^* mice subjected to sham or hepatic I/R (*n* = 8). (E) Representative H&E staining images in liver tissues from *Ubc9^fl/fl^* and *Ubc9^HKO^* mice subjected to sham or hepatic I/R (*n* = 8). Scale bar = 20 or 100 µm. (F) Quantification of necrotic areas in livers from *Ubc9^fl/fl^* and *Ubc9^HKO^* mice subjected to sham or hepatic I/R (*n* = 8). (G) The Suzuki scores of liver sections in *Ubc9^fl/fl^* and *Ubc9^HKO^* mice subjected to sham or hepatic I/R (*n* = 8). (H) Serum ALT/AST levels in *Ubc9^fl/fl^* and *Ubc9^HKO^* mice subjected to sham or hepatic I/R (*n* = 8). IRI6h: ischaemia for 1 h followed by reperfusion for 6 h. All the data are presented as the mean ± SEM. Unpaired Student's *t*‐test was used in (B), (C), (F) and (G). One‐way analysis of variance (ANOVA) was used in (D) and (H). ^**^ *p *< .01; ^***^ *p *< .001; ns, not significant.

To explore the role of Ubc9 in hepatic I/R injury, we established hepatocyte‐specific *Ubc9* transgenic (*Ubc9* ^*Tg*^) and hepatocyte‐specific *Ubc9* KO (*Ubc9^HKO^*) mouse models. Western blot results confirmed *Ubc9* deficiency and overexpression in the livers (Figure S3A, B). The above mice were undergone 1 h period of hepatic ischaemia, followed by 6 h of reperfusion to induce liver I/R damage. Analysis using haematoxylin and eosin (H&E) staining revealed that *Ubc9^Tg^* mice exhibited significantly decreased edema, sinusoidal congestion, vacuolisation, hepatocellular necrosis and sinusoidal haemorrhage compared to NTg mice (Figure 2A–C). And *Ubc9^Tg^* mice exhibited significantly decreased serum ALT and AST levels compared with that in NTg mice (Figure 2D). Furthermore, *Ubc9^HKO^* mice demonstrated pronounced oedema, sinusoidal congestion, vacuolisation, hepatocellular necrosis and sinusoidal haemorrhage (Figure 2E, F). And *Ubc9^HKO^* mice also displayed increased liver injury during hepatic I/R, as manifested by increased Suzuki scores along with increased ALT and AST serum levels than that in *Ubc9^fl/fl^* mice (Figure 2G, H). Collectively, these findings imply that Ubc9 ameliorates liver I/R damage.

### 3.3 Ubc9 ameliorates I/R‐induced inflammation

**FIGURE 3:**
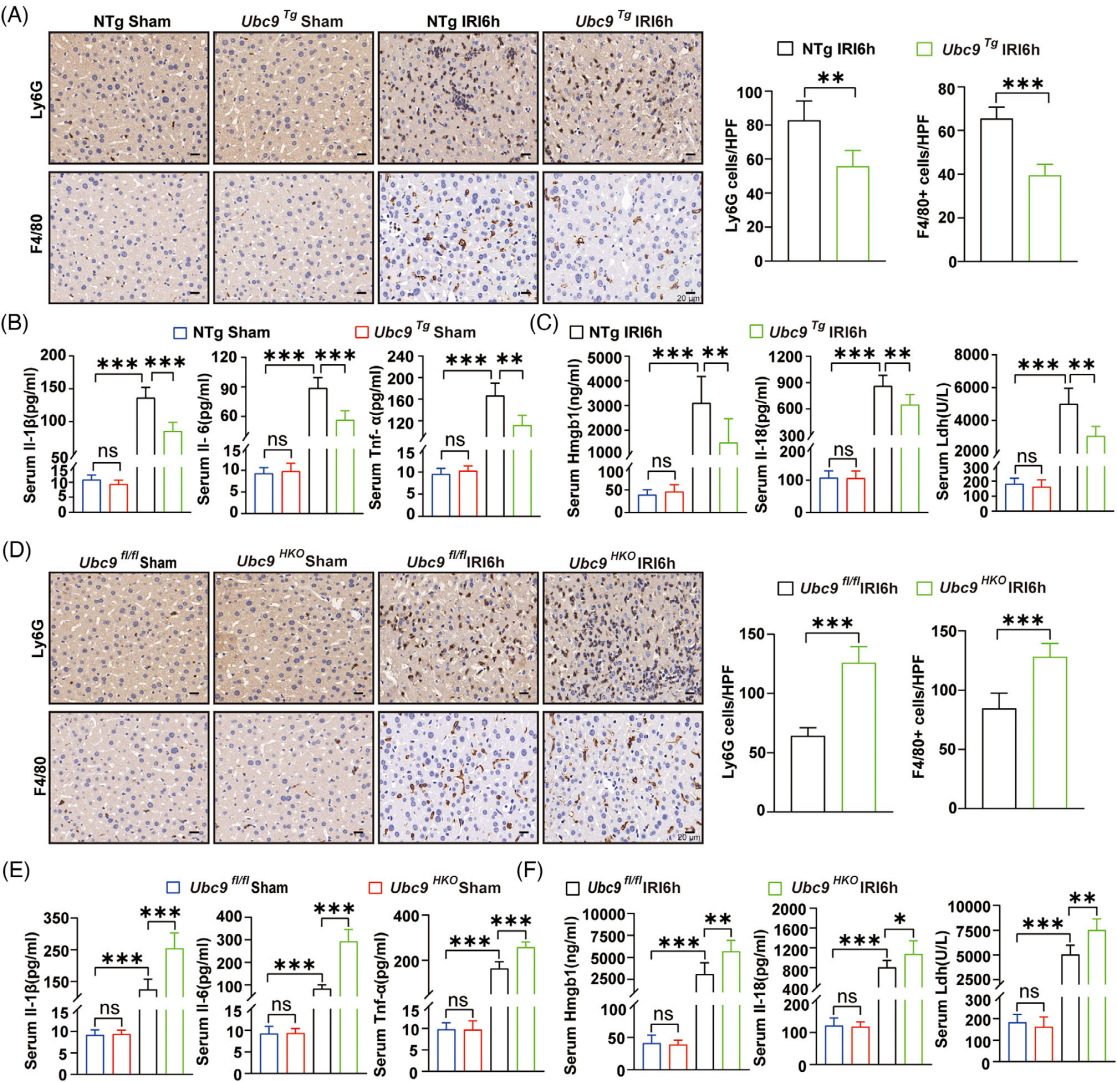
Ubc9 inhibits ischaemia/reperfusion (I/R)‐induced liver inflammation. (A) Representative immunohistochemical (IHC) staining and quantification of F4/80^+^ and Ly6G^+^ cell accumulation in livers of NTg or *Ubc9^Tg^* mice subjected to sham or hepatic I/R (*n* = 8). Scale bar = 20 µm. (B) Levels of serum pro‐inflammatory cytokines (Il‐1β, Il‐6 and Tnf‐α) in NTg and *Ubc9^Tg^* mice subjected to sham or hepatic I/R (*n* = 8). (C) Detection of Hmgb1, Il‐18 and Ldh in serum in NTg and *Ubc9^Tg^* mice subjected to sham or hepatic I/R (*n* = 8). (D) Representative IHC staining and quantification of F4/80^+^ and Ly6G^+^ cell accumulation in livers of *Ubc9^fl/fl^* and *Ubc9^HKO^* mice subjected to sham or hepatic I/R (*n* = 8). Scale bar = 20 µm. (E) Levels of serum pro‐inflammatory cytokines (Il‐1β, Il‐6 and Tnf‐α) in *Ubc9* ^fl/fl^ and *Ubc9* ^HKO^ mice subjected to sham or hepatic I/R (*n* = 8). (F) Detection of Hmgb1, Il‐18 and Ldh in serum in *Ubc9^fl/fl^* and *Ubc9^HKO^* mice subjected to sham or hepatic I/R (*n* = 8). IRI6h: ischaemia for 1 h followed by reperfusion for 6 h. All the data are presented as the mean ± SEM. Unpaired Student's *t*‐test was used in (A) and (D). One‐way analysis of variance (ANOVA) was used in (B), (C), (E) and (F). ^*^ *p *< .05; ^**^ *p *< .01; ^***^ *p *< .001; ns, not significant.

Sterile inflammation represents a hallmark in liver I/R.^1^ Consequently, we investigated the relationship between Ubc9 and inflammatory responses in liver I/R. IHC staining indicated a substantial decrease in Ly6G^+^ neutrophils and F4/80^+^ macrophages in *Ubc9^Tg^* mice livers compare to NTg mice livers (Figure 3A). This observation was further supported by flow cytometry analysis, which showed decreased proportions of neutrophils and macrophages among NPCs in *Ubc9^Tg^* mice livers than that in NTg mice livers following I/R insult (Figure S4A). Furthermore, *Ubc9^Tg^* mice displayed dramatically lower serum and hepatic mRNA levels of *Il‐1β*, *Il‐6* and *Tnf‐α* than that in NTg mice (Figures 3B and S4B). In line with these observations, we found that *Ubc9^Tg^* mice had lower serum levels of Hmgb1, Il‐18 and Ldh than that in NTg mice after hepatic I/R (Figure 3C). In sharp contrast, IHC staining of neutrophils and macrophages showed a markedly higher number of inflammatory cells in *Ubc9^HKO^* mice livers than in *Ubc9^fl/fl^* mice livers (Figure 3D). Consistently, flow cytometry analysis of neutrophils and macrophages from NPCs further had a number of inflammatory cells in *Ubc9^HKO^* mice livers than in *Ubc9^fl/fl^* mice livers (Figure S4C). Moreover, serum and hepatic mRNA levels of *Il‐1β*, *Il‐6* and *Tnf‐ɑ* were increased in *Ubc9^HKO^* mice compared to *Ubc9^fl/fl^* mice (Figures 3E and S4D). Serum Hmgb1, Il‐18 and Ldh were also increased in *Ubc9^HKO^* mice compared to *Ubc9^fl/fl^* mice (Figure 3F). Collectively, our findings demonstrate that Ubc9 attenuates liver inflammatory responses following hepatic I/R.

### 3.4 Ubc9 regulates hepatic DAMPs release and macrophage activation in AML12 cells H/R

**FIGURE 4:**
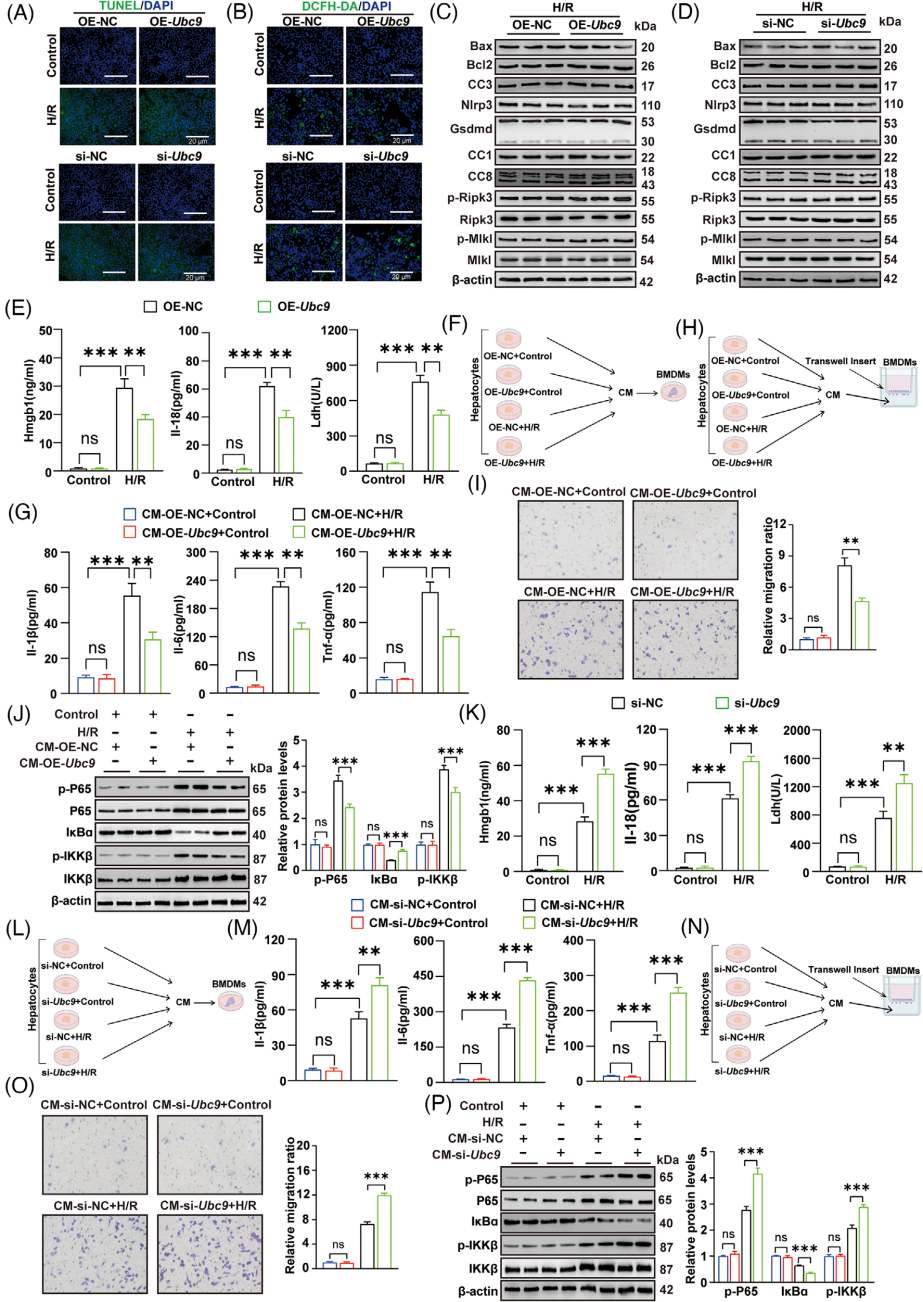
Ubc9 alleviates inflammatory responses after hypoxia/reoxygenation (H/R) treatment. (A) TUNEL staining in OE‐*Ubc9* or si‐*Ubc9* AML12 cells after H/R‐IRI6h: ischaemia for 1 h followed by reperfusion for 6 h. treatment (*n* = 3). Scale bar = 20 µm. (B) Reactive oxygen species (ROS) production was measured using the DCFH‐DA in OE‐*Ubc9* or si‐*Ubc9* AML12 cells after H/R treatment (*n* = 3). Scale bar = 20 µm. (C) Western blot analysis of apoptosis, pyroptosis and necroptosis markers in OE‐NC or OE‐*Ubc9* AML12 cells after H/R treatment. CC3, cleaved caspase3; CC1, cleaved caspase1; CC8, cleaved caspase8 (*n* = 3). (D) Western blot analysis of apoptosis, pyroptosis and necroptosis markers in si‐NC or si‐*Ubc9* AML12 cells after H/R treatment. CC3, cleaved caspase3; CC1, cleaved caspase1; CC8, cleaved caspase8 (*n* = 3). (E) Hmgb1, Il‐18 and Ldh in culture supernatants were measured in OE‐NC or OE‐*Ubc9* AML12 cells after H/R treatment (*n* = 3). (F) Schematic representation of the co‐culture of bone marrow‐derived macrophages (BMDMs) and OE‐NC or OE‐*Ubc9* AML12 cells. (G) Il‐1β, Il‐6 and Tnf‐ɑ in culture supernatants were measured by ELISA in BMDMs stimulated by conditioned medium (CM) from OE‐NC or OE‐*Ubc9* AML12 cells (*n* = 3). (H) Schematic representation of transwell experiment in BMDMs treated by CM from OE‐NC or OE‐*Ubc9* AML12 cells. (I) Transwell migration assay in BMDMs stimulated by CM from OE‐NC or OE‐*Ubc9* AML12 cells (*n* = 3). (J) Western blot analysis of nuclear factor‐kappa B (NF‐κB) signalling pathway in BMDMs stimulated by CM from OE‐NC or OE‐*Ubc9* AML12 cells (*n* = 3). Phosphorylated proteins (p‐P65 and p‐IKKβ) were normalised to relative total proteins (P65 and IKKβ). IκBɑ was normalised to β‐actin. (K) Hmgb1, Il‐18 and Ldh in culture supernatants were measured in si‐NC or si‐*Ubc9* AML12 cells after H/R treatment (*n* = 3). (L) Schematic representation of the co‐culture of BMDM and si‐NC or si‐*Ubc9* AML12 cells. (M) Il‐1β, Il‐6 and Tnf‐ɑ in culture supernatants were measured by ELISA in BMDMs stimulated by CM from si‐NC or si‐*Ubc9* AML12 cells (*n* = 3). (N) Schematic representation of transwell experience in BMDMs treated by CM from si‐NC or si‐*Ubc9* AML12 cells. (O) Transwell migration assay in BMDMs stimulated by CM from si‐NC or si‐*Ubc9* AML12 cells (*n* = 3). (P) Western blot analysis of NF‐κB signalling pathway in BMDMs stimulated by CM from si‐NC AML12 or si‐*Ubc9* AML12 cells (*n* = 3). Phosphorylated proteins (p‐P65 and p‐IKKβ) were normalised to total proteins (P65 and IKKβ). IκBɑ was normalised to β‐actin. All the data are presented as the mean ± SEM. One‐way analysis of variance (ANOVA) was used in (E), (G), (I–K), (M), (O) and (P). ^**^ *p *< .01; ^***^ *p *< .001; ns, not significant.

To investigate the function of Ubc9 in hepatocytes during hepatic I/R and to exclude the effect of other cells on hepatocytes, in vitro H/R model was utilised to replicate in vivo hepatic I/R. We established *Ubc9*‐overexpression and *Ubc9*‐knockdown AML12 hepatocyte cell lines, as confirmed by Western blot (Figure S5A, B). From cell morphology, *Ubc9*‐knockdown AML12 hepatocyte cells developed bubble‐like herniation during H/R treatment, but *Ubc9*‐overexpression AML12 hepatocyte cells mainly exhibited the swollen morphology during H/R treatment (Figure S5C). The bubble‐like herniation of plasma membrane is an obvious indicator of plasma membrane rupture (PMR) and swollen morphology was related to PMR dysfunction.^18^, ^28^ Generally, the pathological process of hepatic I/R injury includes overproduction of ROS, cell death and excessive inflammation responses. Notably, upon H/R challenge, *Ubc9*‐overexpression or *Ubc9*‐knockdown did not significantly altered the proportion of TUNEL‐positive apoptotic cells relative to OE‐NC or si‐NC under H/R conditions (Figures 4A and S5D). Similarly, measurement of ROS production using 2',7'‐Dichlorodihydrofluorescein Diacetate (DCFH‐DA) Macrophage Colony‐Stimulating Factor staining revealed no significant differences in *Ubc9*‐overexpression or *Ubc9*‐knockdown cells compared to OE‐NC or si‐NC after H/R challenge (Figures 4B and S5E). Furthermore, apoptosis, pyroptosis and necroptosis markers remained unchanged in *Ubc9*‐overexpression or *Ubc9*‐knockdown compared to OE‐NC or si‐NC after H/R challenge (Figures 4C, D and S5F,G).

The findings above led us to investigate the effect of Ubc9 on hepatic inflammatory responses. The Il‐1β, Il‐6 and Tnf‐ɑ in the culture supernatants was also remained unaltered in *Ubc9*‐overexpression or *Ubc9*‐knockdown cells compared to OE‐NC or si‐NC after H/R challenge (Figure S5H, I). As the release of DAMPs from injury and death of hepatocytes are associated with a cascade of inflammatory responses, which further aggravates liver damage.^29^ Thus, our aims were to explore the impact of Ubc9 on DAMPs release such as Hmgb1, Il‐18 and Ldh. *Ubc9*‐overexpression in hepatocytes showed significant decreased levels of Hmgb1, Il‐18 and Ldh in the medium after H/R challenge (Figure 4E). Macrophages, being the primary inflammatory cells in the livers, are essential for initiating inflammatory responses to DAMPs.^8^ We further investigated whether hepatocyte Ubc9‐mediated DAMPs release modulates macrophage activation. To this aim, we collected CM from H/R‐treated hepatocytes to stimulate BMDMs (Figure 4F). BMDMs treated with CM from *Ubc9*‐overexpression hepatocytes induced by H/R treatment significantly decreased the Il‐1β, Il‐6 and Tnf‐ɑ in the culture medium of BMDMs compared to CM from OE‐NC group (Figure 4G). Transwell assays indicated that the migratory capacity of BMDMs was significantly inhibited by CM from *Ubc9*‐overexpression hepatocytes induced by H/R treatment compared to CM from OE‐NC group (Figure 4H, I), which might results from decreased DAMPs release by *Ubc9* overexpressing hepatocytes following H/R injury. Nuclear factor‐kappa B (NF‐κB) signalling is critical mediator of macrophage activation.^30^ Indeed, we found that CM from *Ubc9*‐overexpression hepatocytes induced by H/R treatment suppressed NF‐κB signalling in BMDMs compared to CM from OE‐NC group (Figures 4J and S5L). In contrast, Hmgb1, Il‐18 and Ldh in the medium were significantly increased in BMDMs treated with CM from *Ubc9*‐knockdown hepatocytes induced by H/R treatment compared to CM from si‐NC group (Figure 4K). Compared to BMDMs treated with CM from si‐NC, BMDMs treated with CM from *Ubc9*‐knockdown AML12 cells induced by H/R treatment exhibited higher levels of Tnf‐ɑ, Il‐1β and Il‐6 in the culture medium (Figure 4L, M). Transwell assays demonstrated that the migratory capacity of BMDMs was significantly increased by CM from *Ubc9*‐knockdown AML12 cells induced by H/R treatment compared to BMDMs treated with CM from si‐NC AML12 cells (Figure 4N, O). Consistently, CM from *Ubc9*‐knockdown AML12 cells induced by H/R treatment promoted NF‐κB signalling in BMDMs (Figures 4P and S5M). Collectively, these findings suggest that Ubc9 in hepatocytes inhibits DAMPs release, thereby suppressing inflammatory responses following hepatic I/R challenge.

**FIGURE 5:**
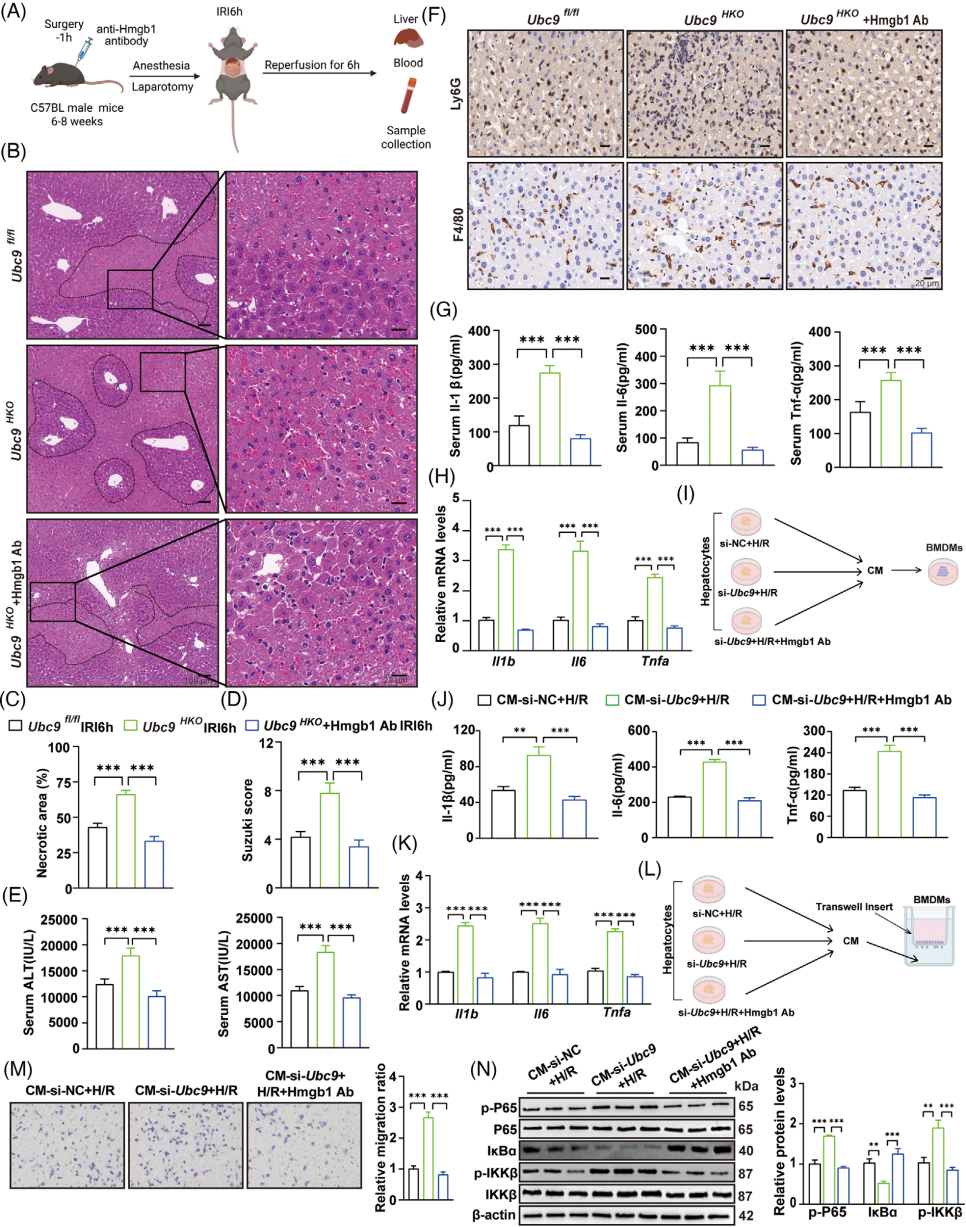
Blocking Hmgb1 reverses the aggravated injury of hepatocyte‐specific *Ubc9* knockout in hepatic ischaemia/reperfusion (I/R)‐induced damage. (A) Schematic of anti‐Hmgb1 neutralisation experiments strategy in hepatic I/R. (B) Representative haematoxylin and eosin (H&E) staining images in livers from *Ubc9^HKO^* mice treated with anti‐Hmgb1 antibody in hepatic I/R (*n* = 8). Scale bar = 20 or 100 µm. (C) Quantification of necrotic areas in livers from *Ubc9^HKO^* mice treated with anti‐Hmgb1 antibody in hepatic I/R (*n* = 8). (D) The Suzuki scores of liver sections in *Ubc9^HKO^* mice treated with anti‐Hmgb1 antibody in hepatic I/R (*n* = 8). (E) Serum alanine aminotransferase (ALT)/aspartate aminotransferase (AST) levels in *Ubc9^HKO^* mice treated with anti‐Hmgb1 antibody in hepatic I/R (*n* = 8). (F) Immunohistochemical (IHC) staining of F4/80^+^ macrophages and Ly6G^+^ neutrophils infiltration in livers from *Ubc9^HKO^* mice treated with anti‐Hmgb1 antibody in hepatic I/R (*n* = 8). Scale bar = 20 µm. (G) Serum levels of Il‐1β, Il‐6 and Tnf‐α were detected by ELISA in *Ubc9^HKO^* mice treated with anti‐Hmgb1 antibody in hepatic I/R (*n* = 8). (H) RT‐qPCR results for pro‐inflammatory genes (*Il‐1β*, *Il‐6* and *Tnf‐α*) expression in livers from *Ubc9^HKO^* mice treated with anti‐Hmgb1 antibody in hepatic I/R (*n* = 8). (I) Schematic representation of co‐culture experiment for conditioned medium (CM) from hepatocytes to stimulate bone marrow‐derived macrophages (BMDMs), or CM plus anti‐Hmgb1 antibody to stimulate BMDMs. (J) Il‐1β, Il‐6 and Tnf‐ɑ in culture supernatants were measured by ELISA in BMDMs stimulated by CM from AML12 cells, or CM plus anti‐HMGB1 antibody (*n* = 3). (K) RT‐qPCR analysis of *Il‐1β*, *Il‐6* and *Tnf‐ɑ* in BMDMs stimulated by CM from AML12 cells, or CM plus anti‐Hmgb1 antibody (*n* = 3). (L) Schematic representation of transwell experiment for CM from AML12 cells to stimulate BMDMs, or CM plus anti‐Hmgb1 antibody to stimulate BMDMs. (M) Transwell migration assay in BMDMs stimulated by CM from AML12 cells to stimulate BMDMs, or CM plus anti‐Hmgb1 antibody to stimulate BMDMs (*n* = 3). (N) Western blot analysis of nuclear factor‐kappa B (NF‐κB) signalling pathway in BMDMs stimulated by CM from AML12 cells, or CM plus anti‐Hmgb1 antibody (*n* = 3). Phosphorylated proteins (p‐P65 and p‐IKKβ) were normalised to relative total proteins (P65 and IKKβ). IκBɑ was normalised to β‐actin. IRI6h: ischaemia for 1 h followed by reperfusion for 6 h. All the data are presented as the mean ± SEM. One‐way analysis of variance (ANOVA) was used in (C–E), (G), (H), (J), (K), (M) and (N). ^**^ *p *< .01; ^***^ *p *< .001.

Extracellular Hmgb1 acknowledged as an significant function in inflammatory responses.^31^, ^32^, ^33^ To further explore that Ubc9 protects mice against I/R injury, Hmgb1 neutralisation antibody was employed (Figure 5A). Hmgb1 neutralisation significantly ameliorated I/R injury caused by *Ubc9* deficiency. Compared with control group, Hmgb1 neutralisation antibody‐treated *Ubc9^HKO^* mice exhibited a markedly reduced hepatic necrosis area, sinusoidal congestion, vacuolisation and sinusoidal haemorrhage, alone with significantly decreased serum AST and ALT levels (Figure 5B–E). IHC staining and flow cytometry analysis showed that Hmgb1 neutralisation attenuated neutrophils and macrophages infiltration in livers from *Ubc9^HKO^* mice (Figures 5F and S6A, B). Inflammatory cytokines profiling by ELISA further corroborated these findings, demonstrating that the elevated levels of Il‐1β, Il‐6 and Tnf‐α were substantially reduced by neutralising Hmgb1 antibody in *Ubc9^HKO^* mice (Figure 5G). Hmgb1 neutralisation also inhibited *Il‐1β*, *Il‐6* and *Tnf‐ɑ* expression of in *Ubc9^HKO^* mice during hepatic I/R (Figure 5H). Similar results of in vitro experiment were obtained in *Ubc9*‐knockdown AML12 cells. Consistently, Hmgb1 neutralisation inhibited the effect of co‐culture medium on BMDMs, as exhibited by the reduced expression and production of Il‐1β, Il‐6 and Tnf‐ɑ (Figure 5I–K). Transwell assays demonstrated that Hmgb1 neutralisation inhibited migratory ability of BMDMs (Figure 5L, M). And Hmgb1 neutralisation suppressed NF‐κB signalling in BMDMs (Figures 5N and S6C).

### 3.5 Ubc9 mediates SUMOylation of Ninj1

**FIGURE 6:**
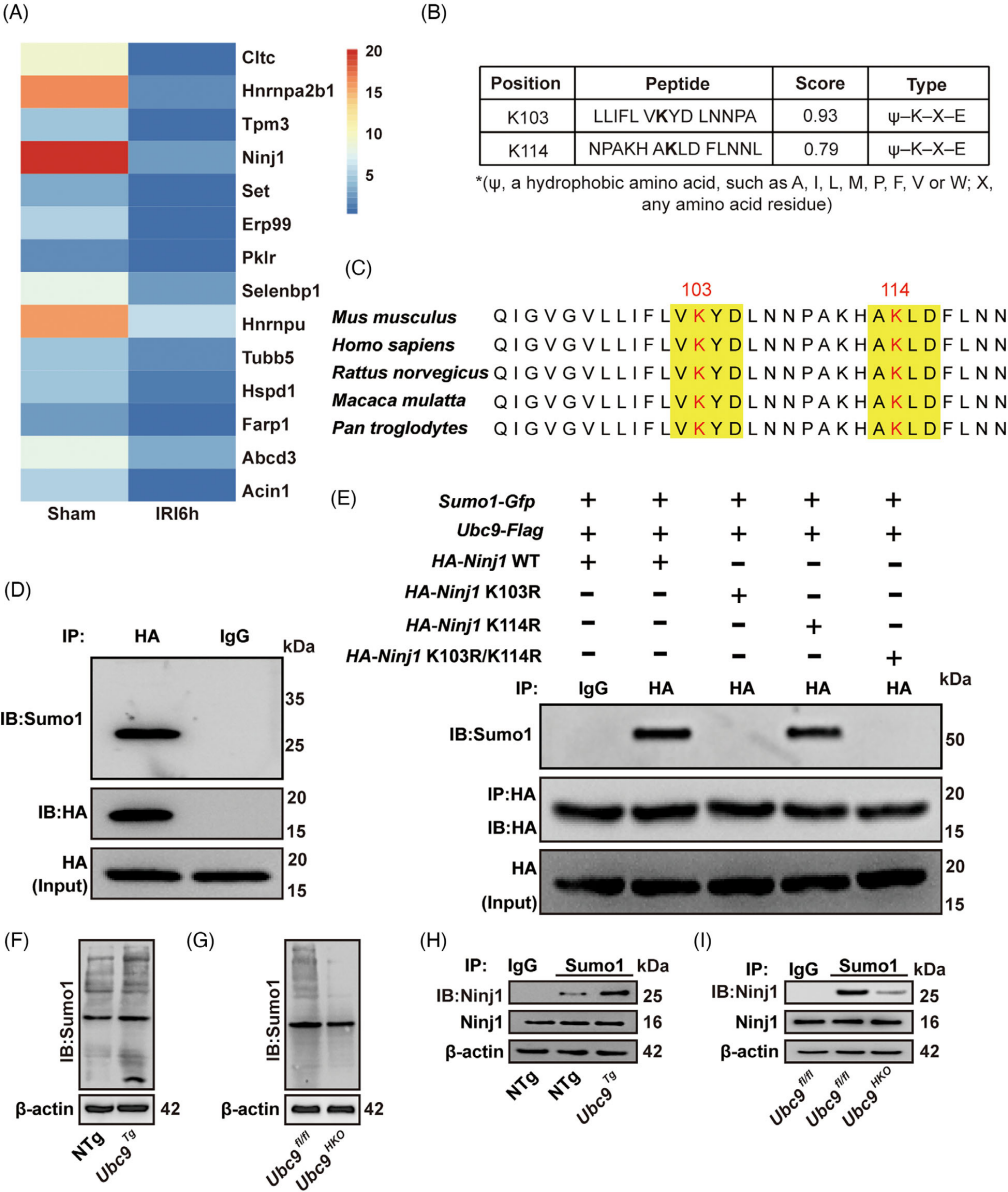
Ubc9 mediates SUMOylation of Ninj1 in hepatic ischaemia/reperfusion (I/R) injury. (A) Heat maps for SUMOylated proteins in mouse liver tissues after sham or hepatic I/R treatment and SUMOylation levels identified by immunoprecipitation and mass spectrometry (IP‒MS). (B) Two sites were predicted as candidate SUMOylation sites on Ninj1. (C) Multiple sequence alignment showing the conservation of residue R103 and R114 of Ninj1 in indicated species. (D) An in vitro SUMOylation assay. HEK293T cells were co‐transfected with *HA*‐*Ninj1* plasmid, *Sumo1* plasmid and *Ubc9* plasmid. Cell lysate was prepared in the presence of 10 mM N‐Ethylmaleimide (NEM), and immunoprecipitated Ninj1‐HA was probed for SUMOylation using an anti‐Sumo1 antibody. (E) Result for in vitro SUMOylation of Ninj1. HEK293T cells were co‐transfected with *HA*‐*Ninj1* wild‐type (WT) plasmid, *HA*‐*Ninj1* K103R plasmid, *HA*‐*Ninj1* K114R plasmid, or *HA*‐*Ninj1* K103R/K114R plasmid, *Sumo*1‐*Gfp* plasmid and *Ubc9*‐*Flag* expression vectors. (F) Sumo1‐conjuncted substrates levels in livers from NTg and *Ubc9^Tg^* mice. (G) Sumo1‐conjuncted substrates levels in livers from *Ubc9^fl/fl^* and *Ubc9^HKO^* mice. (H) SUMOylation of Ninj1 was assessed in the livers of NTg and *Ubc9^Tg^* mice. (I) SUMOylation of Ninj1 was assessed in livers of *Ubc9^fl/fl^* and *Ubc9^HKO^* mice.

To further get a comprehensive insight into the function of Ubc9, we conducted immunoprecipitation and mass spectrometry to analyse Ubc9‐mediated SUMOylation substrates in liver I/R injury. In the identified SUMOylation substrates (Figure 6A), Ninj1 is one of the most highest change of SUMOylation substrates, and Ninj1 regulates PMR and DAMPs release.^18^ Kayagaki et al. demonstrated that hepatic I/R injury increased serum levels of DAMPs (Hmgb1 and Il‐18) in a Ninj1‐dependent manner, which was then selected for further study. Subsequently, we intended to identify SUMOylation sites in Ninj1.^28^ Two lysine residues (K103 and K114) as the possible SUMOylation sites in Ninj1 predicted by the SUMOplot Analysis tools (https://www.abcepta.com.cn/sumoplot; https://sumo.biocuckoo.cn/) (Figures 6B and S7A, B). These two sites are highly conserved among different species (Figure 6C). To verify that Ninj1 can be modified by SUMOylation, *HA*‐tagged WT *Ninj1* plasmids were next co‐transfected with *Sumo1* and *Ubc9* into HEK293T cells. Indeed, the results indicated that Ninj1 could be SUMOylated (Figure 6D). To ascertain the SUMOylation sites of Ninj1, we generated lysine‐to‐arginine substitution mutants of *Ninj1*, including *Ninj1* K103R, *Ninj1* K114R and *Ninj1* K103R/K114R. These mutants, *Gfp*‐tagged *Sumo1* and *Flag*‐tagged *Ubc9*, were transfected into HEK293T cells and performed IP assays as described above, respectively. Indeed, K103 is the SUMOylation site, as evidenced by the absence of SUMOylated *Ninj1* in the K103R or *Ninj1* K103R/K114R transfected cells (Figure 6E). And overexpression of *Ubc9* significantly increased the total Sumo1‐conjugated proteins in the liver (Figure 6F). However, KO of *Ubc9* significantly inhibited the level of the total Sumo1‐conjugated proteins (Figure 6G). Consistently, Ninj1 exhibited higher SUMOylation levels in *Ubc9^Tg^* mice liver than NTG mice liver (Figure 6H). Conversely, the result showed that SUMOylation levels of Ninj1 were lower in *Ubc9^HKO^* mice compared to *Ubc9^fl/fl^* mice (Figure 6I).

### 3.6 SUMOylation of Ninj1 at K103 mediates the protective effect of Ubc9 against I/R‐induced hepatic injury

**FIGURE 7:**
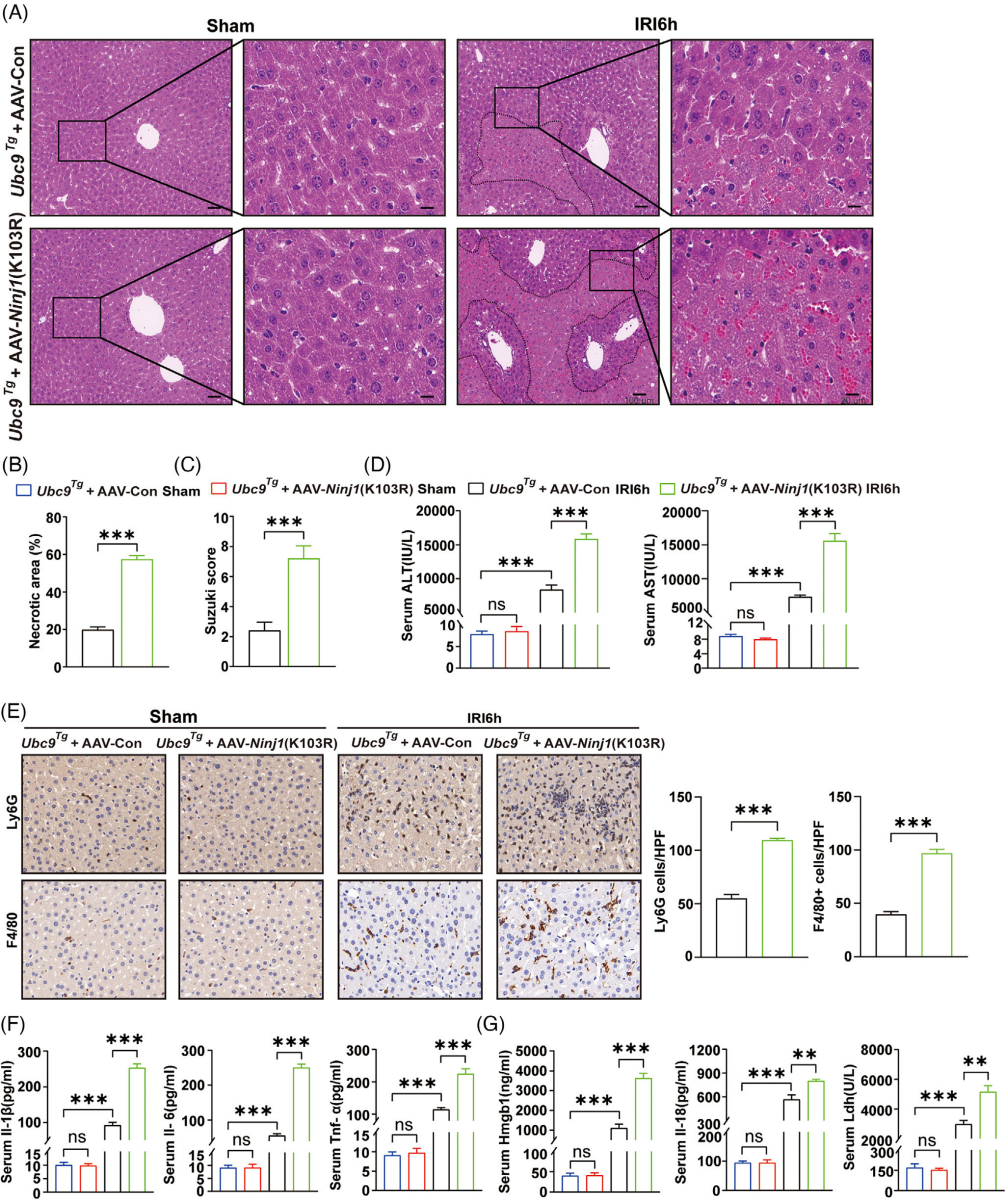
SUMOylation of Ninj1 at K103 mediates the protective effect of Ubc9 against ischaemia/reperfusion (I/R)‐induced hepatic injury. (A) Representative haematoxylin and eosin (H&E) staining images in liver tissues from *Ubc9^Tg^* mice treated with AAV‐Con or AAV‐*Ninj1*(K103R) subjected to sham or hepatic I/R (*n* = 6). Scale bar = 20 or 100 µm. (B) Quantification of necrotic areas in livers from *Ubc9^Tg^* mice treated with AAV‐Con or AAV‐*Ninj1*(K103R) subjected to sham or hepatic I/R (*n* = 6). (C) The Suzuki scores of liver sections in *Ubc9^Tg^* mice treated with AAV‐Con or AAV‐*Ninj1*(K103R) subjected to sham or hepatic I/R (*n* = 6). (D) Serum alanine aminotransferase (ALT)/aspartate aminotransferase (AST) levels in *Ubc9^Tg^* mice treated with AAV‐Con or AAV‐*Ninj1*(K103R) subjected to sham or hepatic I/R (*n* = 6). (E) Immunohistochemical (IHC) staining of F4/80^+^ macrophages and Ly6G^+^ neutrophils infiltration in livers from *Ubc9^Tg^* mice treated with AAV‐Con or AAV‐*Ninj1*(K103R) subjected to sham or hepatic I/R (*n* = 6). (F) Serum levels of Il‐1β, Il‐6 and Tnf‐α were detected by ELISA in *Ubc9^Tg^* mice treated with AAV‐Con or AAV‐*Ninj1*(K103R) subjected to sham or hepatic I/R (*n* = 6). (G) Detection of Hmgb1, Il‐18 and Ldh in serum in *Ubc9^Tg^* mice treated with AAV‐Con or AAV‐*Ninj1*(K103R) subjected to sham or hepatic I/R (*n* = 6). IRI6h: ischaemia for 1 h followed by reperfusion for 6 h. All the data are presented as the mean ± SEM. Unpaired Student's *t*‐test was used in (B), (C) and (E). One‐way analysis of variance (ANOVA) was used in (D), (F) and (G). ^**^ *p *< .01; ^***^ *p *< .001; ns, not significant.

To explore whether Ubc9 exerts its protective effects specifically through SUMOylation of Ninj1 at K103, we used AAV‐*Ninj1* K103R to overexpressed the K103R Ninj1 mutant in *Ubc9^Tg^* mice. The protective effect of Ubc9 was significantly counteracted by AAV‐*Ninj1* K103R in hepatic I/R, as demonstrated by a significant increase in necrotic area, Suzuki scores and serum ALT and AST levels (Figure 7A–D). IHC staining demonstrated a significant reduction in Ly6G^+^ neutrophils and F4/80^+^ macrophages in the livers of *Ubc9^Tg^* mice, which could be reversed by AAV‐Ninj1 K103R (Figure 7E). Additionally, decreased serum levels of Il‐1β, Il‐6, Tnf‐α, Hmgb1, Il‐18 and Ldh in *Ubc9^Tg^* mice were also found to be reversible by AAV‐Ninj1 K103R (Figure 7F,G). Collectively, these findings indicate that the SUMOylation of Ninj1 at lysine 103 plays a crucial role in mediating the protective effect of Ubc9 against hepatic I/R injury.

### 3.7 Ubc9‐mediated Ninj1 SUMOylation at K103 is essential for regulating the subcellular distribution of Ninj1 and DAMPs release

**FIGURE 8:**
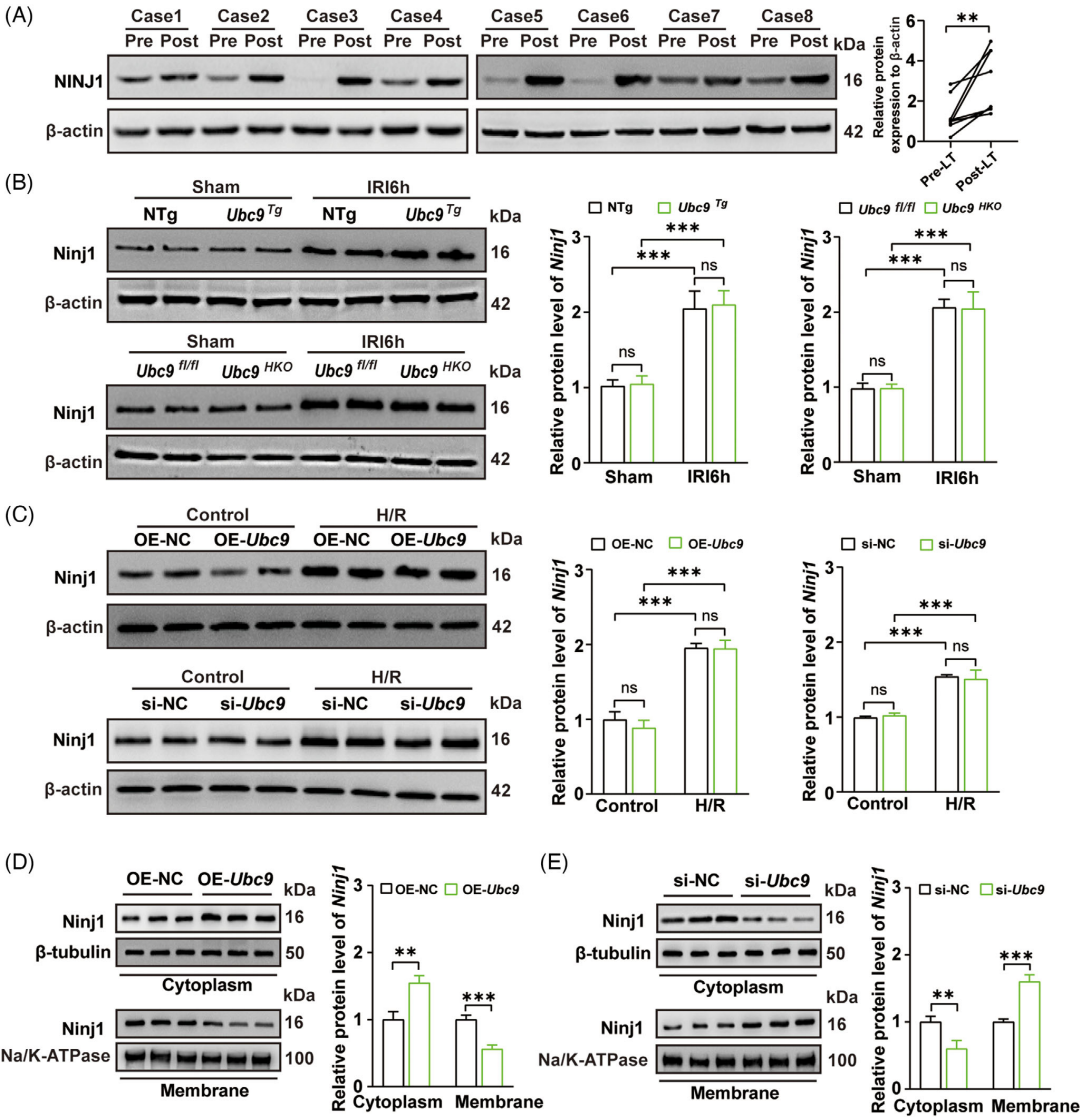
Ubc9 mediates SUMOylation of Ninj1 to inhibit its membrane localisation. (A) Western blot analysis of NINJ1 protein expression in human liver pre‐transplant and post‐transplant tissues (*n* = 8). (B) Western blot analysis of Ninj1 protein expression in livers from *Ubc9^HKO^* or *Ubc9^Tg^* mice and their counterpart control mice subjected to sham or hepatic ischaemia/reperfusion (I/R) (*n* = 8). (C) Western blot analysis of Ninj1 protein expression in OE‐*Ubc9* or si‐*Ubc9* AML12 cells and their counterpart control cells subjected to control or hypoxia/reoxygenation (H/R) (*n* = 4). (D) Subcellular localisation of Ninj1 in OE‐NC or OE‐*Ubc9* AML12 cells subjected to control or H/R (*n* = 3). Cytosol Ninj1 was normalised to β‐tubulin. Membrane Ninj1 was normalised to Na^+^/K^+^ATPase. (E) Subcellular distribution of Ninj1 in si‐NC or si‐*Ubc9* AML12 cells subjected to control or H/R (*n* = 3). Cytosol Ninj1 was normalised to β‐tubulin. Membrane Ninj1 was normalised to Na^+^/K^+^ATPase. IRI6h: ischaemia for 1 h followed by reperfusion for 6 h. All the data are presented as the mean ± SEM. Paired Student's *t*‐test was used in (A). One‐way analysis of variance (ANOVA) was used in (B) and (C). Unpaired Student's *t*‐test was used in (D) and (E). ^**^ *p *< .01; ^***^ *p *< .001; ns, not significant.

To further investigate the effect of Ubc9‐mediated Ninj1 SUMOylation, we first examined Ninj1 expression. The expression levels of NINJ1 were found to be notably elevated in post‐transplant liver samples compared to pre‐transplant samples (Figure 8A). Ninj1 expression was also markedly increased after hepatic I/R injury compared to controls. While hepatocyte‐specific *Ubc9* KO or overexpression did not affect total protein levels of Ninj1 during hepatic I/R injury (Figure 8B, C). Ninj1 is a key mediator of PMR, which facilitates DAMPs release and subsequent immune cell activation.^18^ Previous evidences indicated that inhibiting Ninj1 or PMR could attenuate cell death‐associated inflammatory responses.^34^ Its role relies on its expression and subcellular localisation on the plasma membrane for proper function.^35^ Next, we examined the membrane and cytosol protein levels of Ninj1. Intriguingly, in AML12 cells, overexpression *Ubc9* decreased plasma membrane localisation of Ninj1 following H/R challenge (Figure 8D). However, knockdown of *Ubc9* led to the increase of plasma membrane localisation of Ninj1 following H/R challenge (Figure 8E). These results suggest that Ubc9 may regulate Ninj1 plasma membrane localisation.

**FIGURE 9:**
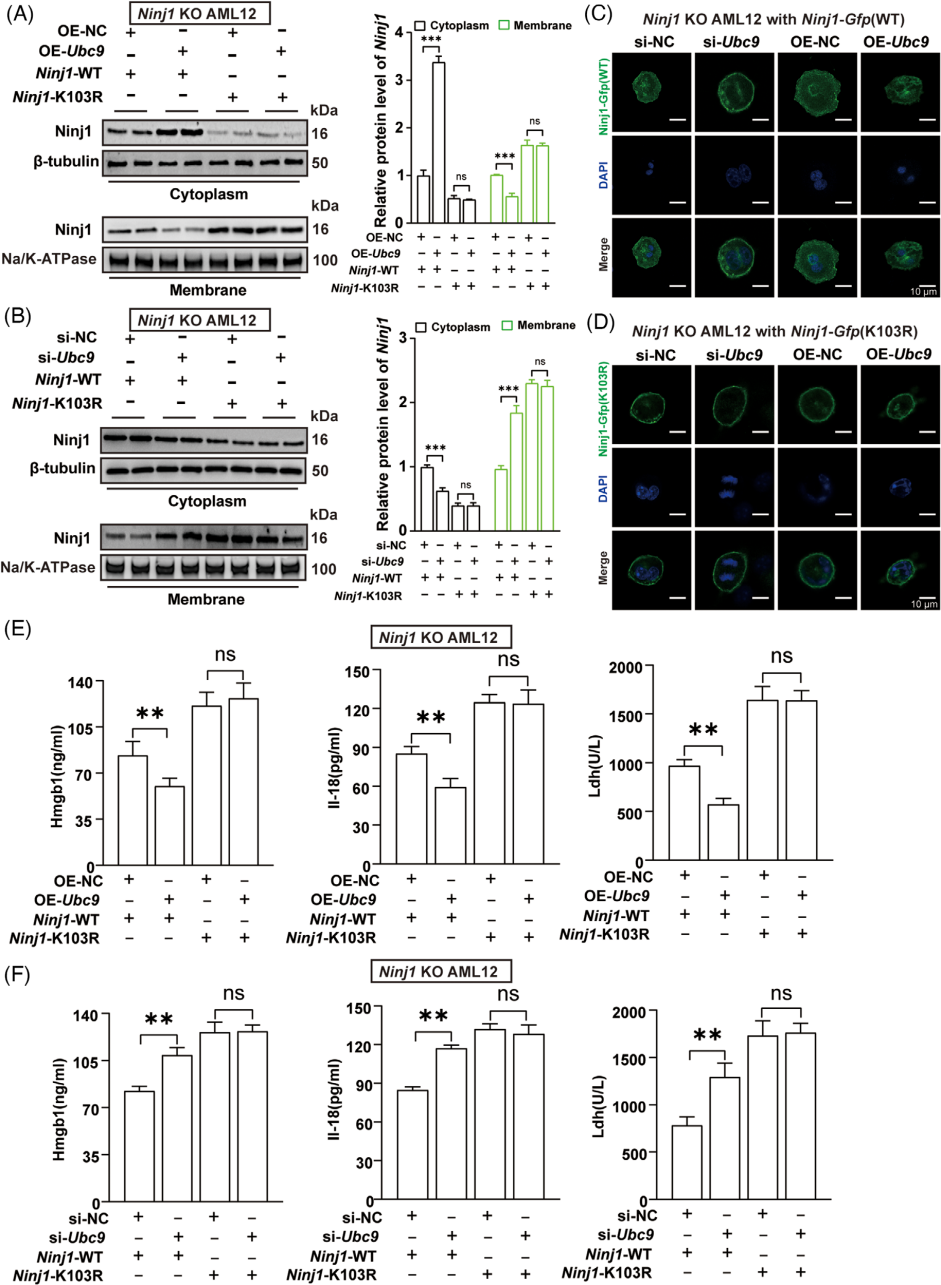
Ubc9 inhibits the plasma membrane localisation of Ninj1 and damage‐associated molecular patterns (DAMPs) release in a Ninj1 K103 SUMOylation‐dependent manner. (A) Distribution of Ninj1 wild‐type (WT) and Ninj1 K103 mutant in OE‐NC or OE‐*Ubc9* AML12 cells subjected to control or hypoxia/reoxygenation (H/R) (*n* = 4). Cytosol Ninj1 was normalised to β‐tubulin. Membrane Ninj1 was normalised to Na^+^/K^+^ATPase. (B) Distribution of Ninj1 WT and Ninj1 K103 mutant in si‐NC or si‐*Ubc9* AML12 cells subjected to control or H/R (*n* = 4). Cytosol Ninj1 was normalised to β‐tubulin. Membrane Ninj1 was normalised to Na^+^/K^+^ATPase. (C) Fluorescence images of *Ninj1* KO AML12 reconstituted with *Ninj1* (WT)‐*Gfp* in H/R‐induced AML12 cells transfected with si‐*Ubc9* or OE‐*Ubc9*. Scale bar = 10 µm. (D) Fluorescence images of *Ninj1* KO AML12 reconstituted with *Ninj1* (K103R)‐*Gfp* in H/R‐induced AML12 cells transfected with si‐*Ubc9* or OE‐*Ubc9* transfection. Scale bar = 10 µm. (E) The levels of Hmgb1, Il‐18 and Ldh in culture supernatants were measured in *Ninj1*‐WT or *Ninj1*‐K103R AML12 transfected with OE‐NC or OE‐*Ubc9* under H/R condition (*n* = 3). (F) The levels of Hmgb1, Il‐18 and Ldh in culture supernatants were measured in *Ninj1*‐WT or *Ninj1*‐K103R AML12 transfected with si‐NC or si‐*Ubc9* under H/R condition (*n* = 3). All the data are presented as the mean ± SEM. One‐way analysis of variance (ANOVA) was used in (A), (B), (E) and (F). ^**^ *p *< .01; ^***^ *p *< .001; ns, not significant.

In order to demonstrate the significance of Ubc9‐mediated Ninj1 SUMOylation at K103 on its subcellular distribution, CRISPR/Cas9 technique was employed to KO endogenous *Ninj1* to generate *Ninj1* KO AML12 cells, which we validated through Western blot and genomic sequencing (Figure S8A, B). We next reconstituted *Ninj1* KO AML12 cells with WT *Ninj1* or K103R *Ninj1* mutation. From cell morphology, overexpression of *Ubc9* mainly exhibited the swollen morphology during H/R treatment in *Ninj1* KO AML12 cells with WT *Ninj1*, but knockdown of *Ubc9* showed bubble‐like herniation during H/R treatment in *Ninj1* KO AML12 cells with WT *Ninj1* (Figure S8C). While overexpression of *Ubc9* or knockdown of *Ubc9* did not perform cell morphology in *Ninj1* KO AML12 cells with K103R *Ninj1* featured by bubble‐like herniation and PMR during H/R treatment (Figure S8D). That suggested Ubc9‐mediated Ninj1 SUMOylation at K103 might play a role in PMR. As expected, overexpression of *Ubc9* decreased WT Ninj1 plasma membrane localisation. In contrast, knockdown of *Ubc9* increased WT Ninj1 plasma membrane localisation (Figure 9A, B). However, overexpression of *Ubc9* or knockdown of *Ubc9* could have no influence on plasma membrane localisation of K103R Ninj1 in H/R challenge (Figure 9A, B). Similarly, fluorescence analysis further confirmed that overexpression of *Ubc9* reduced the plasma membrane localisation of *Gfp*‐tagged WT *Ninj1*, but knockdown of *Ubc9* increased the plasma membrane localisation of *Gfp*‐tagged WT *Ninj1* (Figure 9C). However, overexpression or knockdown of *Ubc9* did not affect the subcellular distribution of *Gfp*‐tagged K103R *Ninj1* mutation during H/R challenge (Figure 9D). Together, these results support that Ubc9‐mediated Ninj1 SUMOylation at K103 is essential for its plasma membrane localisation.

Finally, we sought to further dissect the role of Ubc9‐mediated Ninj1 K103 SUMOylation in DAMPs release such as Hmgb1. Remarkably, overexpression of *Ubc9* decreased the release of Hmgb1, Il‐18 and Ldh in the medium of WT *Ninj1* AML12 under H/R condition (Figure 9E). Furthermore, knockdown of *Ubc9* increased the release of Hmgb1, Il‐18 and Ldh in the medium of WT *Ninj1* AML12 cells under H/R conditions (Figure 9F). However, *Ubc9* did not affect the release of Hmgb1, Il‐18 and Ldh in the medium of K103R *Ninj1* AML12 cells under H/R conditions (Figure 9E, F). Collectively, these results indicate Ubc9 inhibits DAMPs release in a Ninj1 K103 SUMOylation‐dependent manner during AML12 cells H/R.

## 4 DISCUSSION

**FIGURE 10:**
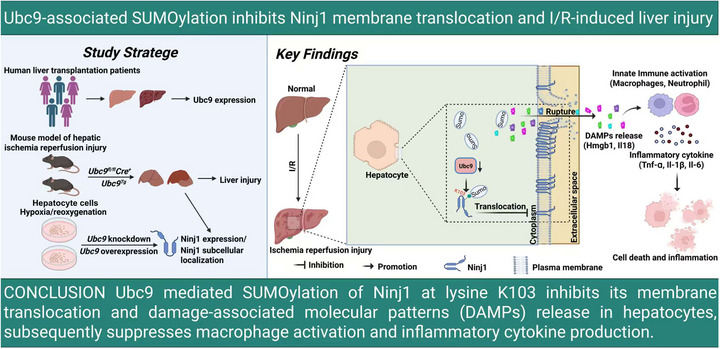
Graphical abstract.

Some investigations have indicated that SUMOylation is pivotal in the response to I/R.^17^, ^36^, ^37^ Thus, our aim is to examine the mechanisms by which SUMOylation influences liver injury associated with I/R. In our research, it was found that pre‐transplant SUMOylation enzyme UBC9 expression in human liver transplants correlated with liver function of patients undergoing LT. Notably, high pre‐transplant UBC9 expression corresponded to better liver function in patients with LT. Meanwhile, we observed a significant reduction in Ubc9 expression in hepatic I/R injury. And Ubc9 was dramatically decrease in hepatocytes, but not in NPCs. We further verified that hepatocyte‐specific *Ubc9* KO aggravated hepatic I/R injury, but hepatocyte‐specific *Ubc9*‐overexpression alleviated hepatic I/R injury. And we identified the Ninj1 SUMOylation on K103. And we found Ubc9‐mediated Ninj1 SUMOylation at K103 is essential for regulating the subcellular distribution of Ninj1 and DAMPs release following hepatic I/R (Figure 10). Thus, this study indicates that Ubc9 might play a protective role in mitigating hepatic I/R injury.

Ubc9 is involved in many critical biological processes. It has been well established that Ubc9 can catalyse the SUMOylation of target proteins to regulate their function.^14^ Specifically, Ubc9 facilitates the SUMOylation of Stat4 at K350, which in turn promotes the ubiquitination‐dependent degradation of Stat4 and serves to inhibit M1 polarisation of macrophages mediated by Stat4.^38^ Ubc9‐induced SUMOylated hnRNPA1 packages Elnat1 into bladder cancer cell‐secreted extracellular vesicles to promote lymph node metastasis in bladder cancer.^39^ Hepatic *SENP1* deficiency SUMOylates RIPK1 in TNF‐R1 signalling complex to aggravate liver inflammation and steatosis in non‐alcoholic steatohepatitis.^40^ Herein, we noted a significant reduction of Ubc9 in liver tissues with patients who underwent LT. UBC9 levels showed an inverse relationship with ALT or AST levels in patients with LT on POD1. Our study demonstrated that Ubc9 expression was significantly decreased after hepatic I/R injury. Our investigation revealed that knockdown or overexpression of *Ubc9* had no significant impact on apoptosis, pyroptosis, necroptosis and oxidative stress. Exploring further, we found that Ubc9 attenuated hepatic inflammatory responses in hepatic I/R injury. As to whether and how Ubc9 might directly or indirectly affect hepatocyte inflammation remains to be elucidated. Knockdown or overexpression of *Ubc9* did not affect Il‐1β, Il‐6 and Tnf‐ɑ in hepatocytes upon H/R treatment. However, we observed that Ubc9 significantly reduced DAMPs (Hmgb1 and Il‐18) and Ldh release during hepatic I/R injury. Hmgb1 is known as one of the most important DAMPs. It is capable of binding to multiple Toll‐like receptors (TLRs), including Tlr2, Tlr4 and Tlr9, thereby initiating inflammatory reactions.^41^, ^42^ Hmgb1 release was also reported in hepatic I/R injury and extracellular Hmgb1 promoted neutrophil extracellular trap and subsequently sterile inflammation.^31^, ^43^ And exaggerated inflammatory response is the main causes of hepatic I/R injury involving two phases: hepatocellular injury and death caused by the interruption of oxygen supply (ischaemia), and followed by a subsequent sterile inflammation upon reperfusion. Neutrophils, macrophages, lymphocytes and dendritic cells (DCs) that play a role in liver I/R injury for sensing damage‐associated signals to prime inflammatory responses.^1^, ^41^ Through sensing DAMPs that are released from dying or dead cells, macrophages recognise early signs of organ injury via pattern recognition receptors (PRRs). This recognition prompts their activation, leading to the release of chemokines and cytokines, which recruit various inflammatory cells, further aggravating liver injury.^8^, ^41^, ^42^ Multiple strategies can influence macrophage activation, such as altering the host microbiota, inhibiting inflammasome signalling pathways, reducing extracellular DAMPs, or blocking DAMP‐receptor signalling in macrophages.^1^, ^28^ Some studies have previously shown that parenchymal hepatocytes contribute to sterile inflammatory responses following hepatic I/R through releasing danger signals such as Hmgb1.^31^, ^32^, ^33^, ^41^, ^44^ Extracellular Hmgb1, as a DAMPs, has been shown to activate directly innate immune cells and initiate sterile inflammatory responses during hepatic I/R injury.^31^, ^41^, ^45^ Indeed, anti‐Hmgb1 neutralising antibodies against Hmgb1 or thrombomodulin (an Hmgb1 inhibitor) have been shown to alleviate hepatocellular injury.^46^, ^47^ Furthermore, macrophages acted a key function in hepatic I/R injury.^4^, ^8^, ^9^ NF‐κB signalling in macrophages is known to promote macrophage activation and inflammation responses.^48^, ^49^ In the study, we found that hepatocyte‐derived Ubc9 could decrease extracellular DAMPs (Hmgb1 and Il‐18) and Ldh following hepatic I/R, inhibit NF‐κB signalling in BMDMs, suppress the inflammatory response, and mitigate liver injury during liver I/R. And anti‐Hmgb1 mAb neutralisation significantly improved liver damage and inflammatory responses associated with KO of Ubc9 during hepatic I/R.

The disintegration of the plasma membrane releases large‐sized cellular contents (known as DAMPs) to the surroundings, which is essential for attracting inflammatory cells and triggering inflammation responses.^35^, ^50^ Recently, it has been known that PMR is a highly regulated process involving Ninj1 protein, rather than a passive osmolysis event.^19^ It acts as an adhesion molecule linked to inflammation and tumor development.^28^, ^51^, ^52^ Ninj1‐mediated PMR facilitates the releases of intracellular DAMPs that activate inflammatory cells.^28^ Our research demonstrated a significant increase of NINJ1 in liver tissues from patients who underwent LT and in mice subjected to hepatic I/R. The inhibition of Ninj1 or the absence of *Ninj1* improved hepatocellular PMR triggered by liver I/R injury.^28^ Ninj1 serves as a vital regulator for mechanical strain‐induced PMR, without necessitating the activation of any cell death pathways.^35^ Ninj1‐dependent PMR is widely found in cells dying by necrosis, apoptosis and pyroptosis.^34^ However, it is noteworthy that Ninj1 is not essential for genetically programmed cell death; still, obstructing Ninj1‐dependent PMR and DAMPs release from dying cells may mitigate the inflammation linked to excessive cell death.^50^ Here, we found Ubc9‐mediated Ninj1 SUMOylation at position 103 (K103). And animal experiments confirmed that the SUMOylation of Ninj1 at lysine 103 (K103) mediates the protective effect of Ubc9 against hepatic I/R injury. Furthermore, we also found that the SUMOylation of Ninj1 at K103 reduced Ninj1 plasma membrane localisation and Hmgb1 and Ldh release in H/R of AML12 cells. However, K103 of Ninj1 was mutated to arginine, knockdown or overexpression of *Ubc9* could not regulate Ninj1 plasma membrane localisation and Hmgb1 and Ldh release in H/R of AML12 cells. Ninj1 is a constitutive membrane protein with a signal sequence, and its membrane localisation is consistent with the hydrophobic nature of ɑ3 and ɑ4 on both the concave and the convex sides of the segment.^20^ Ninj1 SUMOylation on K103 is located near the transmembrane domains of Ninj1. Ninj1 K103 site is located in ɑ3 subunit structure. Ninj1 SUMOylation site might affect the signal sequence to regulate Ninj1 membrane localisation. These results indicates that Ninj1 SUMOylation on K103 is critical for regulating Ninj1 level on the plasma membrane and DAMPs release in H/R of AML12 cells.

In summary, the study provides heretofore unexplored evidence supporting that Ubc9 acts as a crucial factor against liver I/R injury. Mechanistically, Ubc9‐mediated SUMOylation of Ninj1 at lysine K103 to inhibit its membrane localisation and DAMPs release in hepatocytes, subsequently suppressed NF‐κB signalling in macrophages and the production of inflammatory cytokines. These findings further suggest Ubc9‐mediated SUMOylation of Ninj1 at lysine K103 could offer a promising strategy for liver protection against I/R injury in clinical settings.

## AUTHOR CONTRIBUTIONS

Qifa Ye, Cong‐Yi Wang, Wei Zhou and Shaojun Ye designed the study. Kang Huang, Li Xu, Shufang Na, Yan Xu and Qiaoyun Liu performed the experiments, analysed data and wrote the manuscript. Kang Huang, Wei Zhou and Shaojun Ye collected human sample. Qifa Ye, Cong‐Yi Wang and Wei Zhou edited the manuscript. All the authors reviewed and approved the final version of the manuscript.

## CONFLICT OF INTEREST STATEMENT

The authors declare they have no conflicts of interest.

## ETHICS STATEMENT

This research included human participants and received approval from the Medical Ethics Committee of Zhongnan Hospital of Wuhan University (2024100K). The animal research was authorised by Zhongnan Hospital of Wuhan University (ZN2023153).

## Supporting information



Additional supporting information can be found online in the Supporting Information section.

## Data Availability

The data that support the findings of this study are available from the corresponding author upon reasonable request.
