# Supplementary material for: Ubc9‐mediated SUMOylation of Ninj1 alleviates inflammatory responses in hepatic ischaemia/reperfusion injury
Source: Clin Transl Med. 2026 May 10;16(5):e70677. doi: 10.1002/ctm2.70677 (PMC13158375; doi:10.1002/ctm2.70677)
Supplement: Supplementary file 1 — Additional supporting information can be found online in the Supporting Information section. [file CTM2-16-e70677-s001.docx]

**Supplemental information**

**
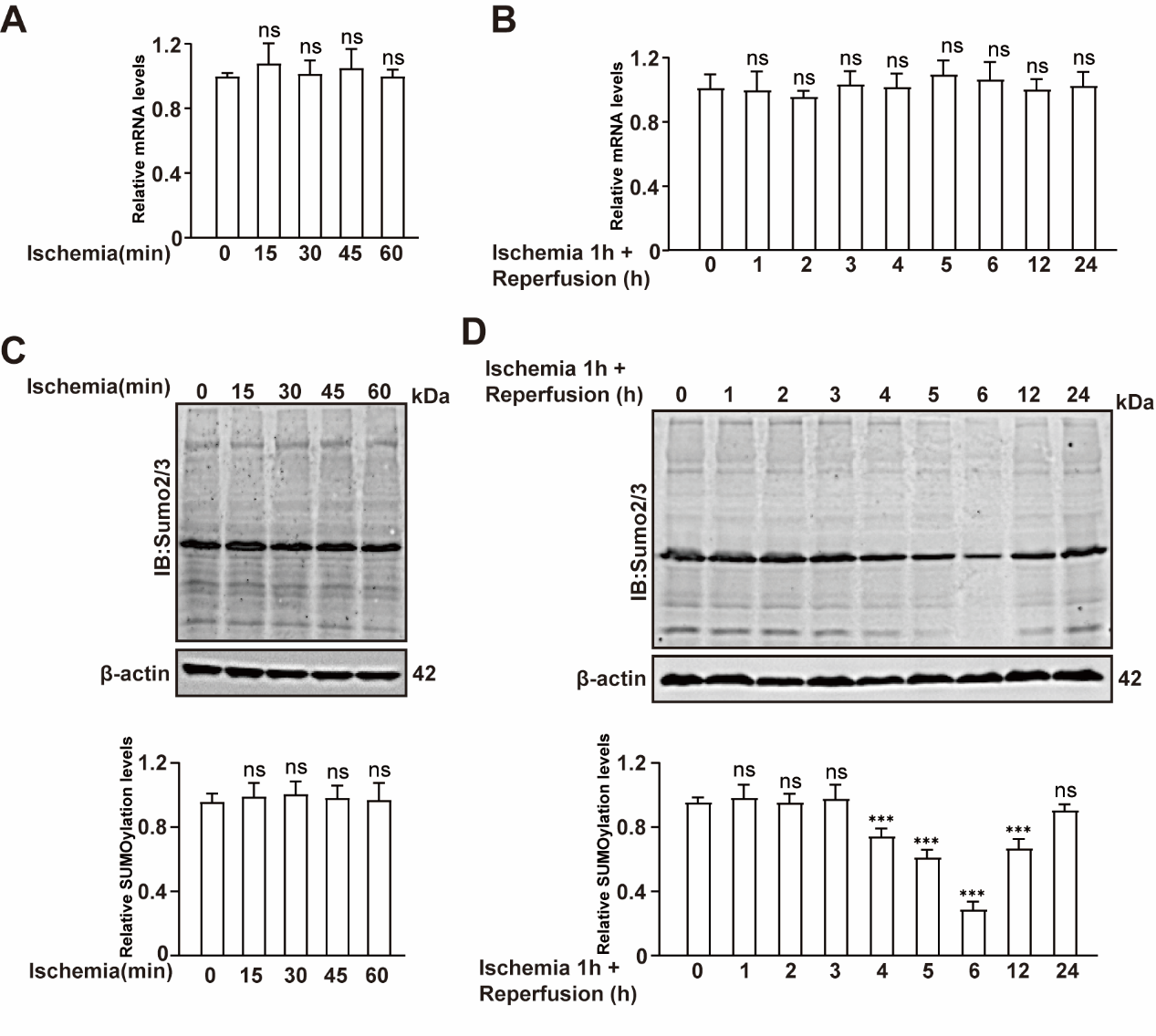
**

**Figure S1 (A)** Ubc9 mRNA levels in livers from mice subjected to sham treatment or ischemia for the indicated times (n = 5). **(B)** Ubc9 mRNA levels in livers from mice subjected to sham treatment or ischemia for 1 hour followed by reperfusion for the indicated times (n = 5). **(C)** Sumo2/3-conjuncted substrates levels in livers from mice subjected to sham treatment or ischemia for the indicated times (n = 5). **(D)** 2/3-conjuncted substrates levels in livers from mice subjected to sham treatment or ischemia for 1 hour followed by reperfusion for the indicated times (n = 5). All date are presented as the mean ± SEM. Unpaired Student’s t-test was used in (A), (B), (C), and (D). ****P* < 0.001; ns, not significant.


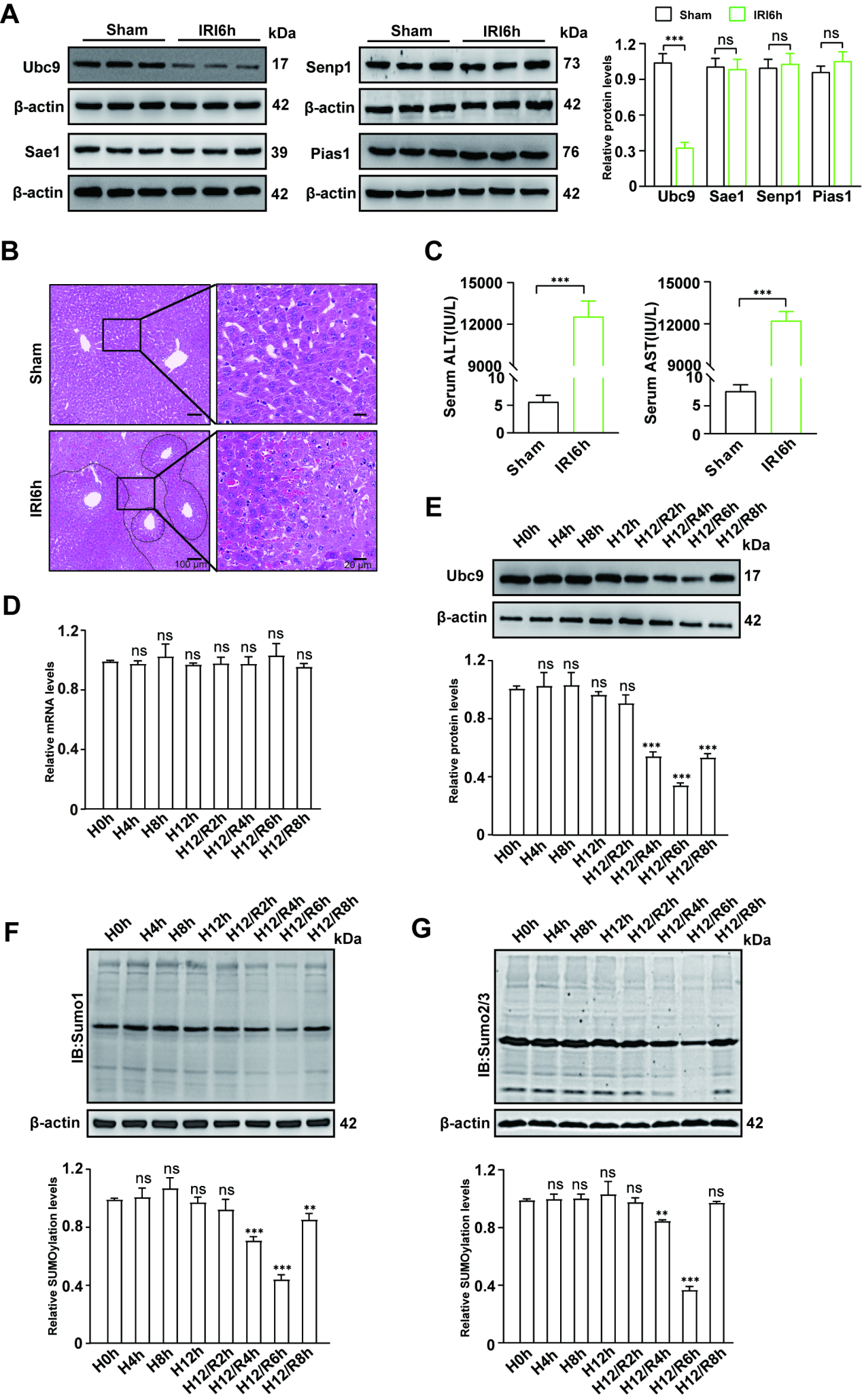


**Figure S2 (A)** Ubc9, Sae1, SenP1, and Pias1 protein levels in livers from mice subjected to sham and hepatic I/R treatment (n = 5). **(B)** Representative H&E staining images from sham and hepatic I/R mice (n = 5). Scale bar = 20 or 100 μm **(C)** Serum ALT/AST levels from sham and hepatic I/R mice (n = 5). **(D)** The Ubc9 mRNA expression levels in AML12 cell lines subjected to H/R treatments for the indicated times (n = 3). **(E)** The Ubc9 protein expression levels in AML12 cell lines subjected to H/R treatments for the indicated times (n = 3). **(F)** Sumo1-conjuncted substrates levels in AML12 cell lines subjected to H/R treatments for the indicated times (n = 3). **(G)** Sumo2/3-conjuncted substrates levels in AML12 cell lines subjected to H/R treatments for the indicated times (n = 3). IRI6h: ischemia for 1 hour followed by reperfusion for 6 hours. H0h, H4h, H8h, H12h: hypoxia for 0 hour, 4 hours, 8 hours, or 12 hours. H12R2h, H12R4h, H12R6h, H12R8h: hypoxia for 12 hours followed by reoxygenation for 2 hours, 4 hours, 6 hours, or 8 hours. All date are presented as the mean ± SEM. Unpaired Student’s t-test was used in (A), (C), (D), (E), (F), and (G). ***P* < 0.01; ****P* < 0.001; ns, not significant.

**
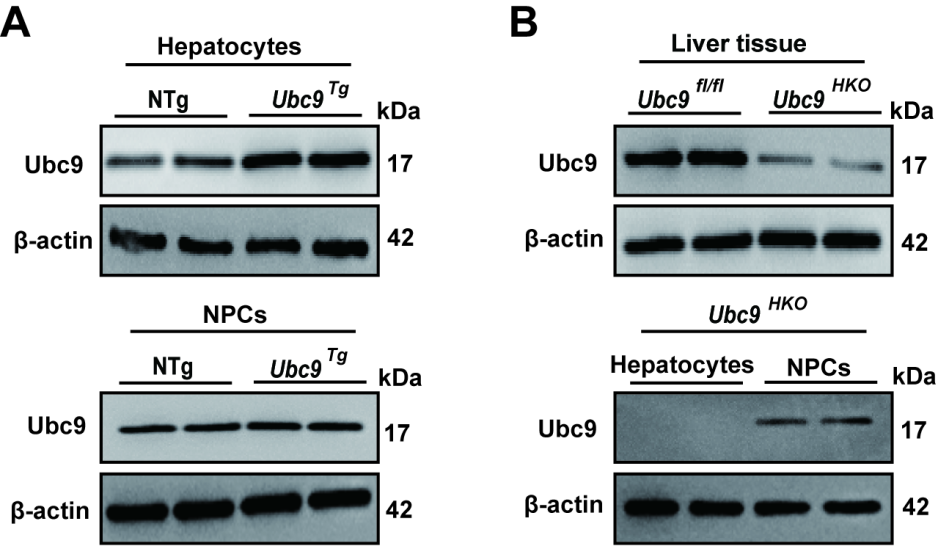
**

**Figure S3 (A)** Western blots for detection of Ubc9 in hepatocytes and NPCs from NTg and *Ubc9^Tg^* mice. **(B)** Western blots for detection of Ubc9 in liver tissues, hepatocytes and NPCs from *Ubc9^fl/fl^* and *Ubc9^HKO^* mice.

**
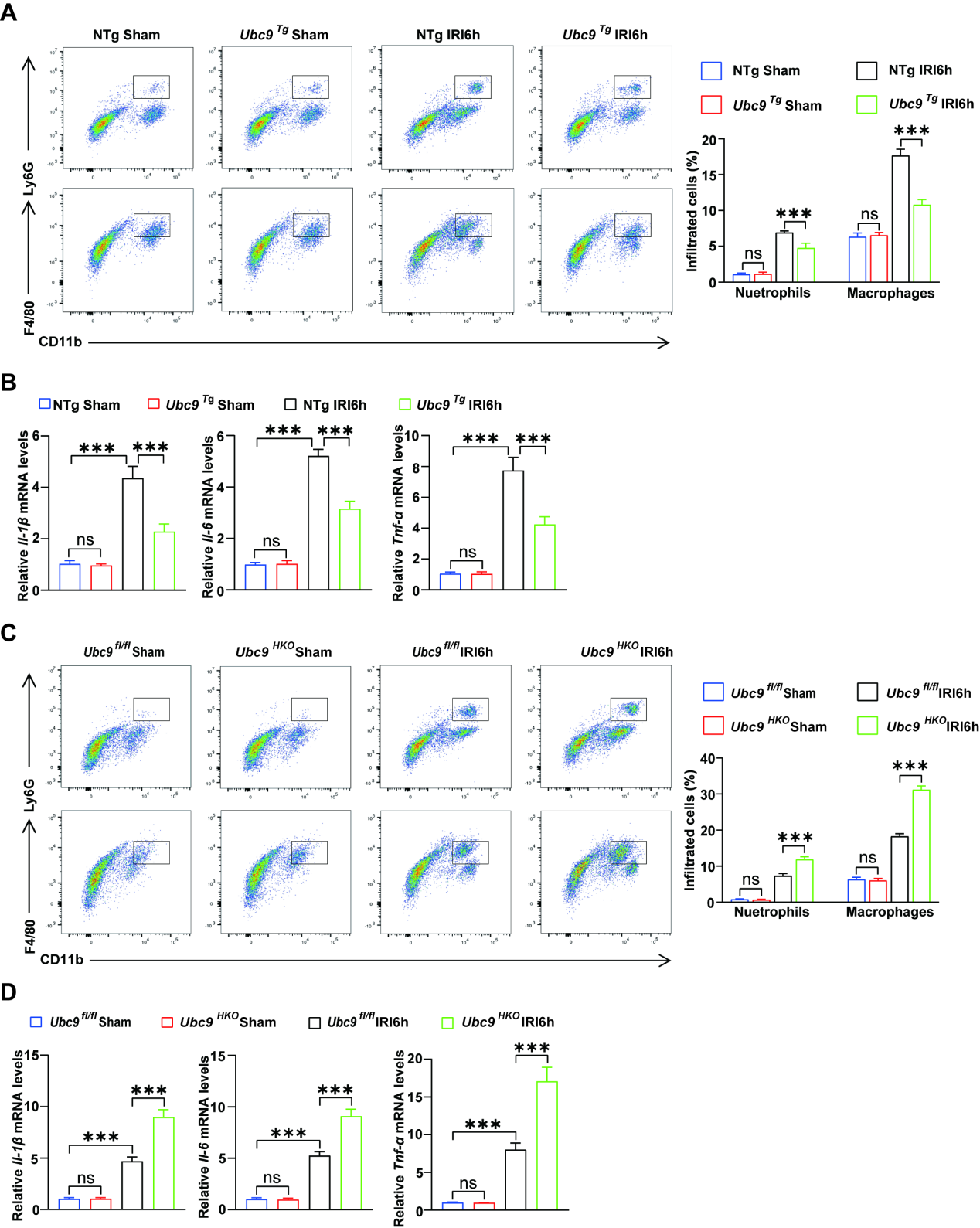
**

**Figure S4 (A)** Flow cytometry analysis was conducted to examine the proportion of neutrophils and macrophages in NTg and *Ubc9^Tg^* mice subjected to sham or hepatic I/R (n = 6). **(B)** mRNA levels of inflammatory cytokines (Il-1β, Il-6, and Tnf-ɑ) in NTg and *Ubc9^Tg^* mice subjected to sham or hepatic I/R (n = 8). **(C)** Flow cytometry analysis was conducted to examine the proportion of neutrophils and macrophages in *Ubc9^fl/fl^* and *Ubc9^HKO^* mice subjected to sham or hepatic I/R (n = 6). **(D)** mRNA levels of inflammatory cytokines (Il-1β, Il-6, and Tnf-ɑ) in *Ubc9^fl/fl^* and *Ubc9^HKO^* mice subjected to sham or hepatic I/R (n = 8). All date are presented as the mean ± SEM. One-way ANOVA was used in (A), (B), (C), and (D). ****P* < 0.001; ns, not significant.

**
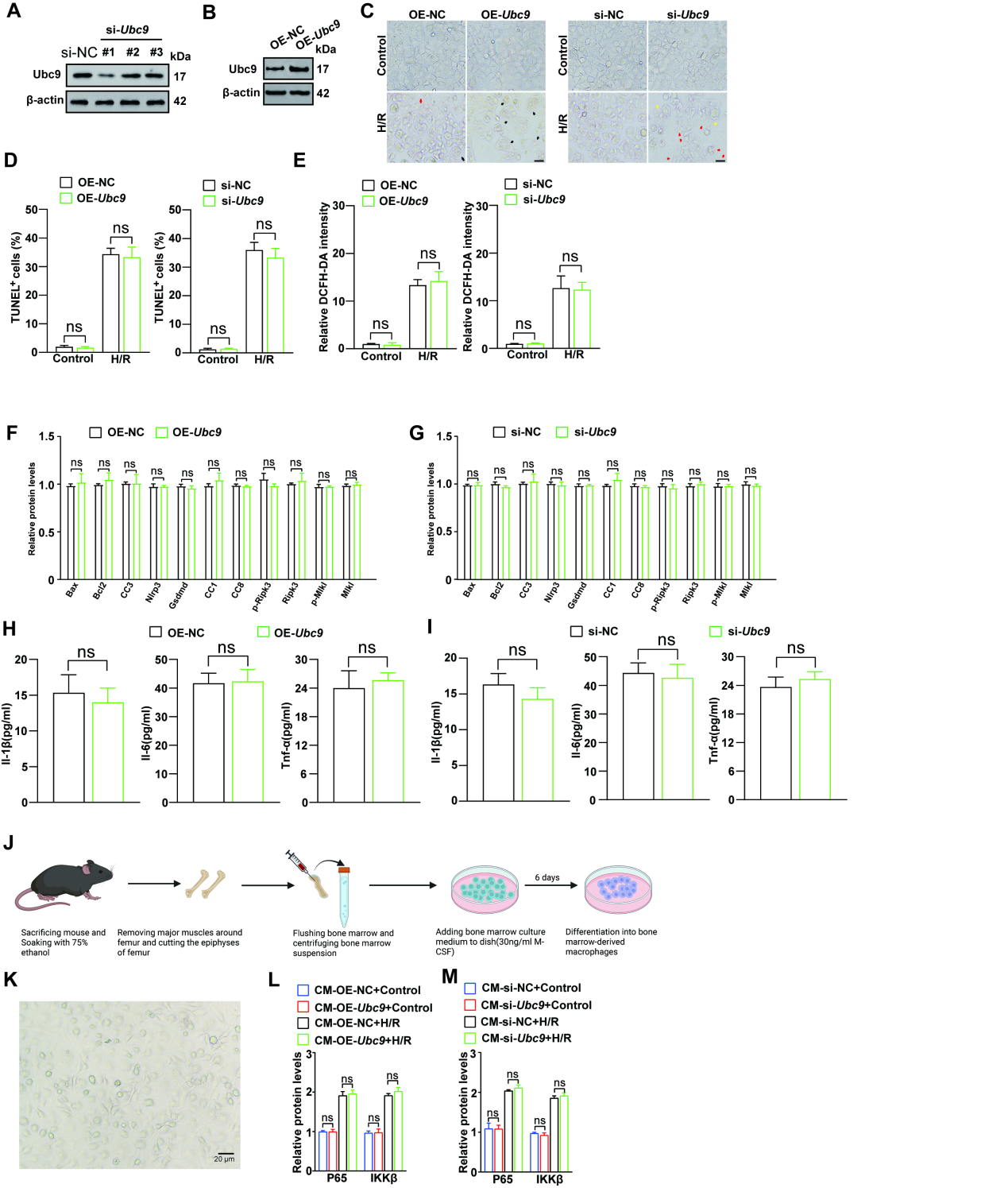
**

**Figure S5 (A)** Western blot analysis of Ubc9 with control siRNA (si-NC) or *Ubc9* siRNA (si-*Ubc9*) transfection in AML12 cells. **(B)** Western blot analysis of Ubc9 with control plasmid (OE-NC) or *Ubc9* plasmid (OE-*Ubc9*) transfection in AML12 cells. **(C)** Bright-field images of OE-*Ubc9* or si-*Ubc9* AML12 cells after H/R treatment. Black arrows indicate swollen cells. Red arrows indicate cells with bubble-like herniations. Yellow arrows indicate plasma membrane rupture. Scale bar = 20 μm. **(D)** Quantification of TUNEL staining in OE-*Ubc9* or si-*Ubc9* AML12 cells after H/R treatment (n = 3). **(E)** Quantification of ROS levels in OE-*Ubc9* or si-*Ubc9* AML12 cells after H/R treatment (n = 3). **(F)** Quantification of western blot analysis of apoptosis, pyroptosis and necroptosis markers in OE-NC or OE-*Ubc9* AML12 cells after H/R treatment (n = 3). Phosphorylated proteins (p-Ripk3 and p-Mlkl) were normalized to relative total proteins (Ripk3 and Mlkl). Other proteins were normalized to β-actin. **(G)** Quantification of western blot analysis of apoptosis, pyroptosis and necroptosis markers in si-NC or si-*Ubc9* AML12 cells after H/R treatment (n = 3). Phosphorylated proteins (p-Ripk3 and p-Mlkl) were normalized to relative total proteins (Ripk3 and Mlkl). Other proteins were normalized to β-actin. **(H)** The levels of inflammatory cytokines (Il-1β, Il-6, and Tnf-ɑ) in AML12 cells transfected with OE-NC or OE-*Ubc9* under H/R condition (n = 3). (No detection in control groups) **(I)** The levels of inflammatory cytokines (Il-1β, Il-6, and Tnf-ɑ) in AML12 cells transfected with si-NC or si-*Ubc9* under H/R condition (n = 3). (No detection in control groups) **(J)** Schematic representation of collection of bone marrow cells and BMDMs induction. **(K)** Images of BMDMs under the microscope. Scale bar = 20 μm **(L)** Quantification of western blot analysis of P65 and IKKβ in BMDMs stimulated by CM from OE-NC or OE-*Ubc9* AML12 cells (n = 3). Total proteins (P65 and IKKβ) were normalized to β-actin. **(M)** Quantification of western blot analysis of P65 and IKKβ in BMDMs stimulated by CM from si-NC AML12 or si-*Ubc9* AML12 cells (n = 3). Total proteins (P65 and IKKβ) were normalized to β-actin. CM: conditioned medium. BMDMs: bone marrow derived macrophages. All date are presented as the mean ± SEM. One-way ANOVA was used in (D), (E), (L), and (M). Unpaired Student’s t-test was used in (F), (G), (H), and (I). ns, not significant.

**
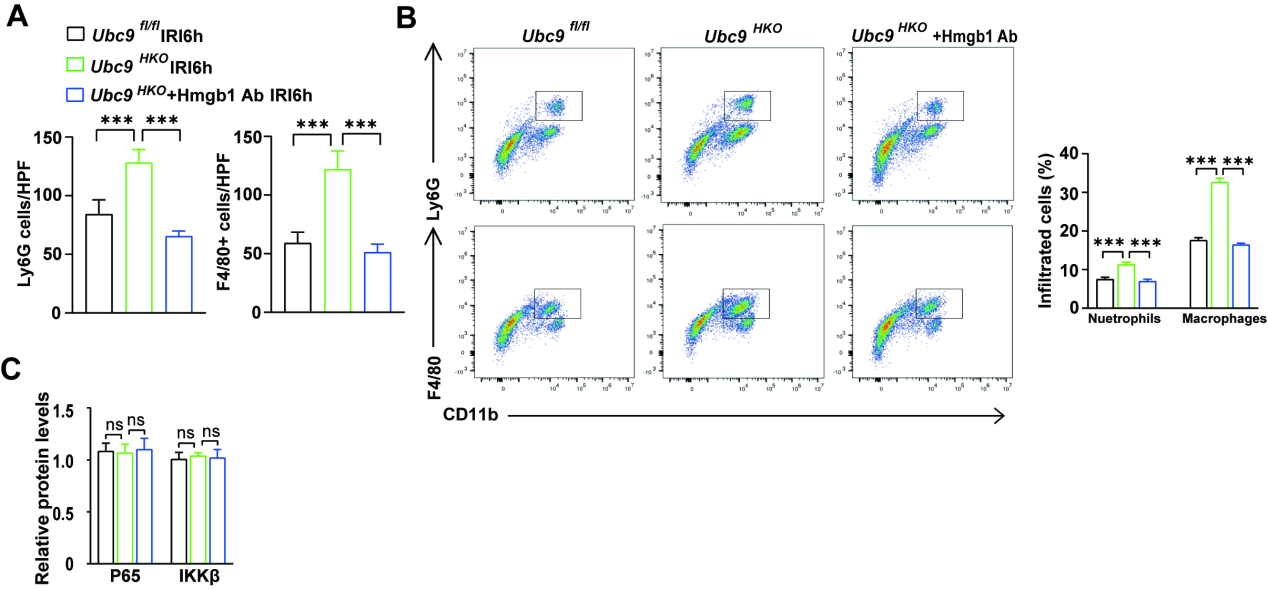
**

**Figure S6 (A)** Quantification of IHC staining of F4/80^+^ macrophages and Ly6G^+^ neutrophils infiltration in livers from *Ubc9^HKO^* mice treated with anti-Hmgb1 antibody in hepatic I/R (n = 8). **(B)** Flow cytometry analysis was conducted to examine the proportion of neutrophils and macrophages in livers from *Ubc9^HKO^* mice treated with anti-Hmgb1 antibody in hepatic I/R. (n = 6). **(C)** Quantification of western blot analysis of P65 and IKKβ in BMDMs stimulated by CM from AML12 cells, or CM plus anti-Hmgb1 antibody (n = 3). Total proteins (P65 and IKKβ) were normalized to β-actin. CM: conditioned medium. BMDMs: bone marrow derived macrophages. All date are presented as the mean ± SEM. One-way ANOVA was used in (A), (B), and (C). ****P* < 0.001; ns, not significant.


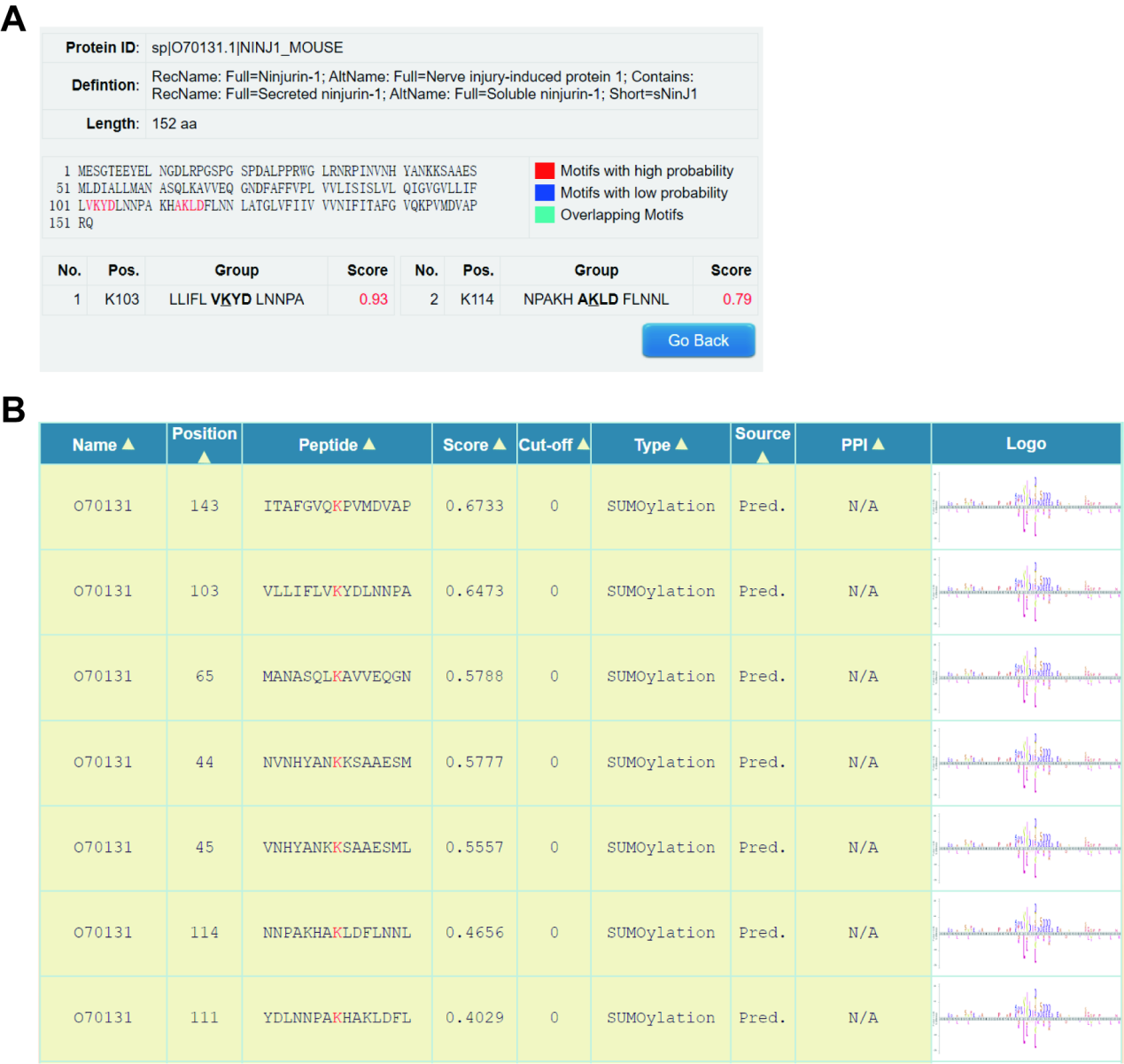


**Figure S7 (A)** SUMOylation site prediction using SUMOplot analysis. **(B)** SUMOylation site prediction using GPS-SUMO analysis.


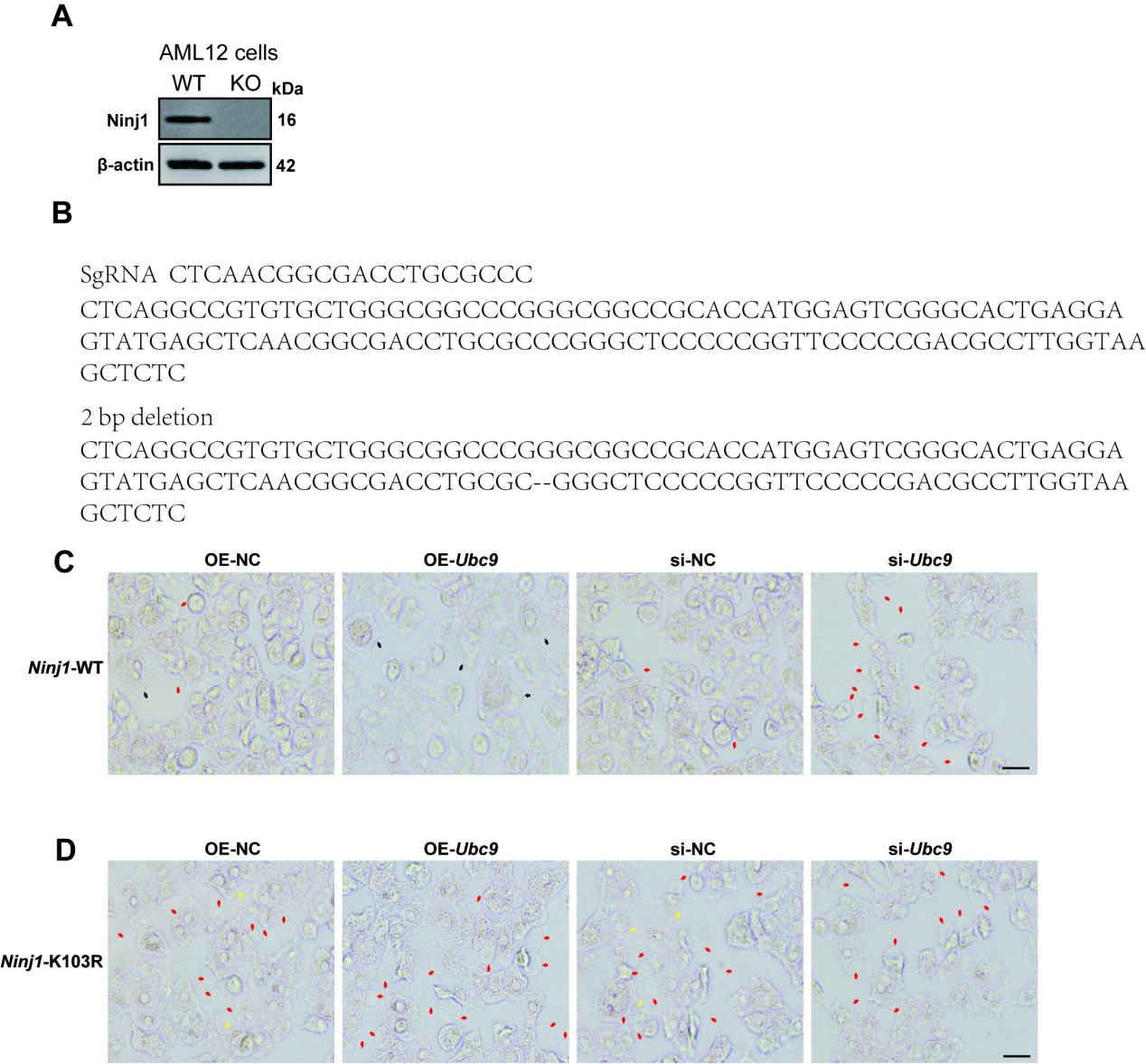


**Figure S8 (A)** Western blot analysis of Ubc9 for WT and KO AML12 cells. **(B)** Genomic sequencing for *Ninj1* KO clone. **(C)** Bright-field images of *Ninj1* KO AML12 reconstituted with *Ninj1*-WT in H/R-induced AML12 cells transfected with si-*Ubc9* or OE-*Ubc9*. Black arrows indicate swollen cells. Red arrows indicate cells with bubble-like herniations. Scale bar = 20 μm. **(D)** Bright-field images of *Ninj1* KO AML12 reconstituted with *Ninj1*-K103R in H/R-induced AML12 cells transfected with si-*Ubc9* or OE-*Ubc9*. Red arrows indicate cells with bubble-like herniations. Yellow arrows indicate plasma membrane rupture. Scale bar = 20 μm.

**Table S1.** **Primers used for RT-qPCR**

| Primer | Sequence5’-3’ |
| --- | --- |
| *Il-1β*-F | CCGTGGACCTTCCAGGATGA |
| *Il-1β*-R | GGGAACGTCACACACCAGCA |
| *Il-6*-F | CCAAGAGGTGAGTGCTTCCC |
| *Il-6*-R | CTGTTGTTCAGACTCTCTCCCT |
| *Tnf-ɑ*-F | CATCTTCTCAAAATTCGAGTGACAA |
| *Tnf-ɑ*-R | TGGGAGTAGACAAGGTACAACCC |
| *β-actin*-F | GTGACGTTGACATCCGTAAAGA |
| *β-actin*-R | GCCGGACTCATCGTACTCC |
| *Ubc9*-F | AGCTGTCCCAACAAAGAACCCT |
| *Ubc9-*R | CACTGTGCCAGAAGGATACACG |

**Table S2. The siRNA sequences**

| siRNA | Sequence |
| --- | --- |
| si*Ubc9*-1 | UGGCACAAUGAACCUGAUGAA(dT)(dT) |
| si*Ubc9*-1 | UUCAUCAGGUUCAUUGUGCCA(dT)(dT) |
| si*Ubc9*-2 | CAGAGUGGAAUAUGAGAAA(dT)(dT) |
| si*Ubc9*-2 | UUUCUCAUAUUCCACUCUG(dT)(dT) |
| si*Ubc9*-3 | CAUCCAAACGUGUAUCCUU(dT)(dT) |
| si*Ubc9*-3 | AAGGAUACACGUUUGGAUG(dT)(dT) |

**Table S3.** **Primers for genotyping**

| Primer | Sequence5’-3’ |
| --- | --- |
| *Ubc9*TypRight_F | TACAGTGCCCACCACCACCATT |
| *Ubc9*TypRight_R | CTCAGGATTCCAGCACCACACG |
| Cre_Typing_F2 | CAGCTAAACATGCTTCATCGTCG |
| Cre_Typing_R | TCCCACCGTCAGTACGTGAGATA |
| InCtrl_F | CAGTAATGCTCCTGTTGTAGCTGCT |
| InCtrl_R | GAGGCGAGTGGTGTTGCTGCT |
| *Ubc9*Tg_Left_F | CGAGGGACCTAATAACTTCGT |
| *Ubc9*Tg_Left_R | GGTTCTTTGTTGGGACAGCT |
| *Ubc9*Tg_Right_F | TCCGAGCAAGCGAAGAA |
| *Ubc9*Tg_Right_R | GACAAGGCTGGTGGGCACT |

**Table S4.** **Donor characteristics stratified by Ubc9 expression of pre-transplant**

| Variables | Low Ubc9 (n = 34) | High Ubc9 (n = 34) | *P* value |
| --- | --- | --- | --- |
| Age (years) | 54.4(14-77) | 58.4(38-79) | 0.195 |
| Gender (M/F) | 27(79.4%)/7(20.6%) | 25(73.5%)/9(26.5%) | 0.568 |
| BMI (kg/m^2^) | 23.3(15.9-27.8) | 22.2(14.2-28.0) | 0.125 |
| Pre-procurement ALT (IU/L) | 25.4(2-103) | 34.2(2-216) | 0.244 |
| Pre-procurement AST (IU/L) | 37.9(6-111) | 35.0(6-133) | 0.632 |
| DBD/DCD | 34/0 | 34/0 | N/A |

M/F: male/female; DBD/DCD: donation after brain death/donation after circulatory death

**Table S5.** **Recipient characteristics stratified by Ubc9 expression of pre-transplant**

| Variables | Low Ubc9 (n = 34) | High Ubc9 (n = 34) | *P* value |
| --- | --- | --- | --- |
| Age (years) | 50(27-71) | 49(31-65) | 0.655 |
| Gender (M/F) | 26(76%)/8(24%) | 23(68%)/11(32%) | 0.418 |
| BMI (kg/m^2^) | 23.8(16.3-30.7) | 25.4(17.7-36.9) | 0.235 |
| Disease etiology |  |  | 0.685 |
| Viral hepatitis B | 12 | 13 |  |
| Viral hepatitis C | 1 | 0 |  |
| EtOH | 3 | 3 |  |
| HCC | 11 | 10 |  |
| AIH | 1 | 4 |  |
| DILI | 2 | 1 |  |
| PLD | 2 | 0 |  |
| Cryptogenic cirrhosis | 2 | 1 |  |
| Wilson’s disease | 0 | 1 |  |
| PBC | 0 | 1 |  |
| ABO compatibility |  |  | N/A |
| Identifical | 34 | 34 |  |
| MELD score | 20.0(7-34) | 20.6(6-46) | 0.803 |
| Pre-transplant ALT (IU/L) | 70.7(12-562) | 76.8(10-1217) | 0.564 |
| Pre-transplant AST (IU/L) | 88.56(14-388) | 106.8(17-927) | 0.562 |
| CIT (min) | 432.2(275-752) | 430.8(104-967) | 0.965 |
| WIT (min) | 0 | 0 | N/A |
| Anhepatic phase (min) | 49.7(36-80) | 50.4(35-108) | 0.834 |
| Recipient operation time (min) | 350.5(225-585) | 322.8(210-555) | 0.144 |
| Intraoperative blood loss (ml) | 1006(100-5000) | 1049(50-6000) | 0.895 |

M/F: male/female; BMI: body mass index; EtOH: ethyl alcohol; HCC：hepatocellular carcinoma; AIH:autoimmune hepatitis; DILI: drug-induced liver injury; PLD: polycystic liver disease; PBC: primary biliary cholangitis; MELD: model for end-stage liver disease; CIT: cold ischemia time; WIT: warm ischemia time

**Supplemental Materials and Methods**

**TUNEL staining and ROS assay**

Terminal deoxynucleotidyl transferase dUTP nick-end labeling (TUNEL) assay was conducted following the instructions provided by the manufacturer (Solarbio, T2196). ROS were evaluated using an ROS Assay Kit (Solarbio, CA1410).

**H&E staining**

Hematoxylin and eosin (H&E) staining performed to assess liver damage. The grading of liver ischemia-reperfusion injury (IRI) followed Suzuki’s criteria, evaluated blindly on a scale from 0 to 4. A score of 0 indicated the absence of hepatocellular necrosis and any signs of congestion or centrilobular ballooning, while a score of 4 was assigned in cases of severe congestion accompanied by more than 60% lobular necrosis. Liver samples were fixed in 4% neutral-buffered formalin (Servicebio, G1101), followed by dehydrated, and embedded in paraffin. Paraffin-embedded liver samples were then sliced into 5-μm-thick continuous slides and subsequently stained with H&E. Images were captured using a light microscope by two pathologists in a blinded manner.

**Immunohistochemical staining**

Hepatic sections that were fixed with formalin and embedded in paraffin were examined using primary rat anti-mouse Ubc9 (ZENBIO, R27399), F4/80 (Cell Signaling Technology, 70076), and Ly6G (Cell Signaling Technology, 87048) mAbs. Positive cells were counted in a blinded manner in at least three fields per section (200×).

**ELISA assay**

Murine serum samples and culture supernatants were gathered for the assessment of Tnf-ɑ (Elabscience, E-MSEL-M0002), Il-1β (Elabscience, E-MSEL-M0003), Il-6 (Elabscience, E-MSEL-M0001), Hmgb1 (Jonlnbio, JL10769), and Il-18 (Jonlnbio, JL20253) concentrations. The concentrations of Tnf-ɑ, Il-1β, Il-6, Hmgb1 and Il-18 in murine serum and culture supernatants were evaluated using enzyme-linked immunosorbent assay (ELISA) kits, in accordance with the guidelines provided by the manufacturer.

**LDH assay**

Lactate dehydrogenase (Ldh) activity in murine serum and culture supernatants were assessed utilizing an LDH Activity Assay Kit (Elabscience, E-BC-K046-M) in accordance with the manufacture’s instructions.

**Flow cytometry analysis**

NPCs were isolated from whole liver (sham) or I/R-insulted liver lobes (1 hour of ischemia followed by 6 hours of reperfusion). Cells were stained with anti-mouse F4/80 (ThermoFisher, 11-4801-85), Ly6G (ThermoFisher, 12-9668-82) and CD11b (ThermoFisher, 45-0112-82) antibodies to identify macrophages and neutrophils infiltration according to the manufacturer’s instructions, respectively. Macrophages were characterized as CD11b+F4/80+, while neutrophils were identified as CD11b+Ly6G+. Following a 30-minute incubation at 37 ℃ in darkness, the suspensions were rinsed with PBS and then re-suspended in 300 μL of PBS. The samples were analyzed and sorted using a CytoFLEX flow cytometer (Beckman Coulter), and the resulting data were processed with FlowJo software (BD Life Sciences).

**Subcellular fraction**

Cells were subjected to extraction of the membrane and cytosol fractions utilizing a protein extraction kit designed for these components (Beyotime, P0033), following the guidelines provided by the manufacturer. The extracted proteins from both the membrane and cytosol were collected for subsequent experiments.

**Immunoprecipitation**

The samples of cells or tissues were collected and lysed after washes with pre-cooled PBS for three times. Immunoprecipitation (IP) was conducted by incubating prepared proteins with indicated antibodies overnight at 4 ℃ with rotation, following cross-linked to protein G (Beyotime, P2053) for an additional 2 hours at 4 ℃. The resulting complexes were washed using IP lysis buffer, and the precipitates were eluted in laemmli buffer (Sigma-Aldrich, R0278) and resolved by SDS-PAGE.
